# Evaluation of logistic regression models and effect of covariates for case–control study in RNA-Seq analysis

**DOI:** 10.1186/s12859-017-1498-y

**Published:** 2017-02-06

**Authors:** Seung Hoan Choi, Adam T. Labadorf, Richard H. Myers, Kathryn L. Lunetta, Josée Dupuis, Anita L. DeStefano

**Affiliations:** 1 Department of Biostatistics, Boston University, 801 Massachusetts Avenue, Boston, Massachusetts USA; 2 Department of Neurology, Boston University, 72 East Concord Street, Boston, Massachusetts USA

**Keywords:** RNA-Sequencing analysis, Firth’s logistic regression, Negative binomial regression, Covariate effect

## Abstract

**Background:**

Next generation sequencing provides a count of RNA molecules in the form of short reads, yielding discrete, often highly non-normally distributed gene expression measurements. Although Negative Binomial (NB) regression has been generally accepted in the analysis of RNA sequencing (RNA-Seq) data, its appropriateness has not been exhaustively evaluated. We explore logistic regression as an alternative method for RNA-Seq studies designed to compare cases and controls, where disease status is modeled as a function of RNA-Seq reads using simulated and Huntington disease data. We evaluate the effect of adjusting for covariates that have an unknown relationship with gene expression. Finally, we incorporate the data adaptive method in order to compare false positive rates.

**Results:**

When the sample size is small or the expression levels of a gene are highly dispersed, the NB regression shows inflated Type-I error rates but the Classical logistic and Bayes logistic (BL) regressions are conservative. Firth’s logistic (FL) regression performs well or is slightly conservative. Large sample size and low dispersion generally make Type-I error rates of all methods close to nominal alpha levels of 0.05 and 0.01. However, Type-I error rates are controlled after applying the data adaptive method. The NB, BL, and FL regressions gain increased power with large sample size, large log2 fold-change, and low dispersion. The FL regression has comparable power to NB regression.

**Conclusions:**

We conclude that implementing the data adaptive method appropriately controls Type-I error rates in RNA-Seq analysis. Firth’s logistic regression provides a concise statistical inference process and reduces spurious associations from inaccurately estimated dispersion parameters in the negative binomial framework.

**Electronic supplementary material:**

The online version of this article (doi:10.1186/s12859-017-1498-y) contains supplementary material, which is available to authorized users.

## Background

Next generation sequencing (NGS) gene expression measurement methods simultaneously quantify tens of thousands of unique Ribonucleic Acid (RNA) molecules extracted from biological samples. These RNA sequencing (RNA-Seq) methods produce data that can be transformed into numerical values that are proportional to the abundance of RNA molecules and reflect the expression and turnover of those molecules. Identifying differentially expressed (DE) genes is an important step to understanding the molecular mechanism of disease. As with any statistical analysis, the underlying structure of the data dictates appropriate methodology. The NGS methods provide a count of RNA molecules that do not follow a normal distribution. The Negative Binomial (NB) distribution appropriately models the biological dispersion of a gene, and NB regression has been used to analyze RNA-Seq data. When Y, a random variable, follows a NB distribution with mean (*μ*) and dispersion (*ϕ*), the parameterization of the probability mass function, expected value, and variance of *Y* are

$$ \begin{array}{l}\kern4em  Y\sim NB\left(\mu, \phi \right),\mathrm{where}\mu \ge 0\mathrm{and}\phi \ge 0\mathrm{suchthat}\\ {} f(y)=\frac{\Gamma \left( y+{\phi}^{-1}\right)}{\Gamma \left({\phi}^{-1}\right)\Gamma \left( y+1\right)}\left(\frac{\phi^{-1}}{\mu +{\phi}^{-1}}\right){}^{\phi^{-1}}\left(1-\frac{\phi^{-1}}{\mu +{\phi}^{-1}}\right){}^y,\\ {}\kern7.5em  E\left[ Y\right]=\mu, \mathrm{andvar}\left[ y\right]=\mu +\mu {\phi}^2.\end{array} $$

Because the total number of reads of each sample will likely be different, a normalization step is required prior to performing differential expression inferences between two conditions. Normalization approaches have been evaluated elsewhere [1]. Based on these evaluations, we implemented the DESeq normalization approach in this study [2].

Estimation of the dispersion parameter (*ϕ*) of each gene is challenging with the small number of observations typically available in RNA-Seq studies. An overestimated dispersion may result in loss of power to detect DE genes and an underestimated dispersion parameter may increase false discoveries. Two of the most sophisticated and widely used software packages for identifying DE genes are DESeq2 and edgeR [3, 4], which estimate the dispersion parameter of each gene using empirical Bayes shrinkage and Cox-Reid adjusted profile likelihood methods, respectively.

Many RNA-Seq analysis methods have been evaluated in different settings including multi-group study designs [1, 5–9]. Soneson et al. [9] compared performance of RNA-Seq analysis tools (edgeR, DESeq, baySeq [10], NBPSeq [11], TSPM [12], EBSeq [13], NOIseq [14], SAMseq [15], ShrinkSeq [16] and limma [17]) using real and simulated data sets. They reported that when sample size is small, the results should be cautiously interpreted. However, when sample size is large, the limma using variance stabilizing transformation method performed well. Seyednasrollah et al. [18] evaluated eight computational methods such as edgeR, DESeq, baySeq, NOISeq, SAMseq, limma, Cuffdiff2 [19], and EBSeq using publically available human and mice RNA-Seq data. They concluded that no method fits for all situations and results from distinct methods could be largely different. Rapaport et al. [7] assessed commonly used analysis packages (Cuffdiff, edgeR, DESeq, PoissonSeq [20], baySeq, and limma) for RNA-Seq data. They analyzed human RNA-Seq data with those methods and emphasize the importance of large sample replicates to accurately detect association with genes. Tang et al. [6] included TCC [21], edgeR, DESeq, DESeq2, voom [22], SAMseq, PoissonSeq, baySeq, and EBSeq in their evaluation with multi-group data. The edgeR and DESeq2 packages were recommended in their assessment. DESeq2 and edgeR are generally accepted in the analysis of RNA-Seq data because these packages are designed to properly handle studies with small sample size and lowly expressed genes. However, the appropriateness of the NB model compared to the logistic model has not been exhaustively evaluated. Because many RNA-Seq studies are designed to compare cases and controls, we explore logistic regression as an alternative approach, in which disease status is modeled as a function of RNA-Seq reads. This is a reversal of the experimental and explanatory variables in the NB model in the RNA-Seq setting. An attractive feature of the logistic framework is that the estimation of a dispersion parameter for gene expression is not necessary. Although some studies analyzed their data modeled as a function of gene expression [23, 24], a comparison to standard NB methods was not conducted. We evaluate logistic regression models in which the dependent variable is disease status and gene expression is the independent variable.

Because of the substantial costs associated with RNA-Seq technology and the challenges in obtaining appropriate tissue sample sizes may be limited in some studies. When sample size is small the distribution of test statistics may not achieve the expected asymptotic distribution. Hence, we incorporated the data adaptive method into NB and logistic regressions because this approach estimates a re-calibrated distribution of test statistics. We evaluated the validity of the data adaptive method in RNA-Seq studies.

Another important feature of differential expression analysis is covariate adjustment. Adjustment for confounders is crucial in protecting against spurious associations. We define a confounder as a covariate that is associated with both the experimental and explanatory variables. Covariates in RNA-Seq analysis are associated with disease status, technical artifacts from experimental methodology, or intrinsic biological properties of a system in RNA-Seq models. If these covariates affect the abundance measurements of RNA-Seq data, then the covariates could significantly confound the association between RNA-Seq and disease status. Again, we considered two approaches for differential expression analysis: 1) NB regression where gene expression values are the outcome variable and case–control status is the predictor variable and 2) logistic regression where case–control status is a function of gene expression values. If disease-associated covariates are not associated with gene expression, then these covariates are non-predictive (NP) covariates in models with RNA-Seq as the outcome. The effect of adjusting for covariates, when the relationship between covariates and gene expression is not assessed through statistical tests or prior studies, has not been extensively evaluated in RNA-Seq studies using the NB framework. If we alternatively consider a logistic model, the NP covariates in the NB model become non-confounding predictive (NCP) covariates in the logistic model, because the covariates are not associated with the independent variable (gene expression) but are associated with the dependent variable (disease status).

We use both simulated data sets and an application to a real Huntington’s disease (HD) RNA-Seq data set [25]. The results of this study will guide the selection of an appropriate regression model and guide decisions regarding covariate adjustment in RNA-Seq studies.

## Methods

This study focuses on a gene as a unit; hence various gene-based scenarios are considered.

### Regression methods for analyzing RNA-Seq data

#### Negative binomial (NB) regression

NB regression uses the Maximum-Likelihood (ML) fitting process [26]. The generalized linear model (GLM) framework is used by DESeq2 and edgeR. In the current study, GLM was implemented using the *glm(,family = negative.binomial(1/ϕ))* function in the R-package “MASS” and utilized either the estimated dispersion from ML, Quasi-likelihood (QL) or the true dispersion value from the simulation scenario. ML and QL methods are described in Additional file 1: Supplementary Method Section 1. In our real data application, the original data and permuted data sets were analyzed with DESeq2 [3].

#### Classical logistic (CL) regression

We conducted GLM in a logistic regression framework using the logit link function using the *glm(,family = binomial)* function in R. In the RNA-Seq setting CL regression may be limited by small sample size and complete separation. If the expression values of a gene are completely or nearly completely separated between case and control groups, which may occur when the effect size is large, the ML estimation from CL regression may fail to converge. Because complete separation may be an indicator of differential expression, we implemented Bayes and Firth’s logistic regressions, which overcome complete separation bias.

#### Bayes logistic (BL) regression

Gelman et al. [27] proposed a prior to estimate stable coefficients in a Bayesian framework, when data show separation. The proposed prior is the Cauchy distribution with center 0 and scale 2.5. They demonstrated that this flat-tailed distribution has robust inference in logistic regression and is computationally efficient. The procedure is implemented by incorporating an EM algorithm into iteratively reweighted least squares, using the *bayesglm* function in the R-package “arm”.

#### Firth’s logistic (FL) regression

The ML estimators may be biased due to small sample size and small total Fisher information. Firth proposed a method that eliminates first-order bias in ML estimation by introducing a bias term in the likelihood function [28]. Heinze and Schemper demonstrated that Firth’s method is an ideal solution when the data show separation [29]. The *logistf* function in the R-package “logistf” was used.

### Simulation study

The performance of statistical models was evaluated through Type-I error and power scenarios using combinations of the parameter values in Table 1.

**Table 1:** Parameters and their values in simulation scenarios

Parameter	Values
Design	Balanced, Unbalanced2, Unbalanced4
Number of cases (*N*_*D=1*_)	10, 25, 75, 500
Mean expression value in controls (*μ*_*D=0*_ *)*	50, 100, 1000, 10000
Dispersion (*ϕ)*	0.01, 0.01, 0.5, 1
Covariate OR (CovOR)	1, 1.2, 3, 5, 10
log_2_ fold-change (log2fc)	0, 0.3, 0.6, 1.2, 2
Number of Covariates	0, 1, 2, 3, 5, 10

#### Generating simulated RNA-Seq data

For each scenario, the read counts (*y*_*i*_) were sampled from the NB distribution with mean (*μ*) and dispersion (*ϕ*) as specified in Table 1. We simulated 10,000 independent replicates per scenario using the following steps and evaluated Type-I error rate and power per scenario based on results from these 10,000 independent replicates.

First, we generated simulated sample (*i*) data with their case (*D = 1*) and control (*D = 0*) statuses determined by the study design shown in Table 1. Then, a gene (*g*) expression value for each sample (*y*_*ig*_) was generated following the NB distribution conditioning on the disease status. The log2fc determined the mean expression value for cases (*y*_*gD=1*_) in power scenarios. When simulating under the null hypothesis (Type-I error scenarios) log2fc = 0 and the mean expression value (*μ*_g*D*_) was equal for cases and controls. We considered only up-regulation of genes, and assumed the dispersion was the same for cases and controls. The simulation model for the RNA-Seq count data is:

$$ {y}_{ig} \sim \mathrm{N}\mathrm{B}\left({\mu}_{g D},{\phi}_g\right), $$

where *μ*_*gD*_ *≥0, ϕ*_*g*_ *≥0, D* is the binary case–control status of a sample (*i*), *μ*_*g*_ is mean expression value of a gene *g. μ*_*gD=1*_ is the mean expression value for cases for gene g and is calculated as *2* ^*log2fc*^ *× μ*_*gD=0*_.

#### Generating simulated covariate data

The binary covariates (***X***) were simulated to follow a binomial distribution conditioning on case–control status of each sample. The conditional probability was calculated based on the CovOR.

$$ X\Big| D \sim \mathrm{B}\left({N}_D,{P}_D\right), $$

where *D* is disease status (control = 0; case = 1), *N*_*D*_ is sample size of *D*, *P*_*D=0*_ = 0.5, and *P*_*D=1*_ *= CovOR/(CovOR + 1)*. When the number of cases is 10, CovOR of 10 is not considered. For every 10 replications among 10,000 replications in a scenario, a new covariate set was generated to incorporate between and within variance of covariates. All covariates in a model were independent and had the same CovOR.

#### Analyzing simulated data

We performed NB regression with Model A and conducted the CL, BL, and FL regressions with Model B.

$$ \begin{array}{l}\kern2em \mathrm{Model}\ \mathrm{A}: \log \left( E\left[ Y\right]\right) = {\beta}_0+{\beta}_1 D\left({\displaystyle {\sum}_{k=1}^C{\beta}_{k+1}{X}_k}\right)\\ {}\mathrm{Model}\ \mathrm{B}:\mathrm{logit}\left( E\left[ D\right]\right) = {\beta}_0^{*}+{\beta}_1^{*} Y+\left({\displaystyle {\sum}_{k=1}^C{\beta}_{k+1}^{*}{X}_k}\right)\end{array} $$

where *Y* is read count, *D* is case/control status and *C* is the number of covariates. Models without covariates were analyzed using all regression methods in order to compare the performances of unadjusted models. Models adjusting for covariates were analyzed only using NB and FL regressions to evaluate different types of covariate effects.

Scenarios for which log2fc = 0 are Type-I error studies. Otherwise, the scenarios are power studies. Type-I error rates, at significance (alpha) levels 0.05 and 0.01, were calculated based on replicates with converged results. For power studies, because different Type-I error rates were observed among the distinct regression methods, comparing the power of different regression methods using the same threshold is not appropriate. For fair comparison, an empirical threshold for each regression method was calculated. Then, we computed the empirical power of each regression method using their empirical thresholds. Although only positive log2fcs were simulated in those scenarios in which power was evaluated, we consistently used two-sided tests for both Type-I error and power studies. The equations for Type-I error rate and empirical power are shown in Additional file 1: Supplementary Method Section 2.

#### Validation of data adaptive (DA) method using cross-validation technique

The DA method re-estimates a distribution of test statistics under the null hypothesis of no association [30]. The DA approach enables one to obtain a recalibrated distribution of test statistics because when sample size is small, the asymptotic distribution may not be appropriate. This method also avoids heavy computing burden compared to implementing permutation tests with all possible permutations. The results from the DA method are validated using a cross-validation technique. Detailed procedures are provided in Additional file 1: Supplementary Method Section 3.

### Huntington’s disease (HD) study

A real RNA-Seq data set was analyzed using the DESeq2 R-package, which implements a NB GLM. The data set was also analyzed utilizing R (v3.0.0) to implement CL, BL, and FL regressions. The logistic regressions modeled case–control status as a function of normalized counts of a gene and covariates. The publicly available HD data set [25] was downloaded from the GEO database (accession number GSE64810, https://www.ncbi.nlm.nih.gov/geo/query/acc.cgi?acc=GSE64810). This data set contains 20 HD cases and 49 neurologically normal controls. Details of the HD data set are provided in Additional file 1: Supplementary Method Section 4.

#### Generating permuted HD Data

The original HD study used RNA Integrity Number (RIN) and Age at Death (AAD) as covariates. RIN was included due to the potential confounding effect between HD and the abundance of RNAs. To remove the effect of RIN in our permutations, at first, samples were divided by RIN categories. Then, each gene was resampled within each category of RIN. Because AAD was included in the regression model due to its association with HD, the relationship between HD and AAD was preserved during the permutation process. We generated 10,000 Monte-Carlo permutations.

#### Analyzing permuted HD data

For the original HD data and each permutated data set, DE genes were identified using the NB model (Model A) as implemented in DESeq2. We also implemented the CL, BL, and FL regressions (Model B) to compare statistical models. In logistic models, we used normalized counts from DESeq2 as an independent variable. For these analyses, we adjusted for AAD and RIN.

The Type-I error rates at our alpha levels using supplementary equation (2.1), and the exact *p*-values [31] were calculated with the results from the 10,000 permutations. The DA method was applied using our permutation results [30] to measure Type-I error rates and to obtain adjusted *p*-values of each gene.

For HD data analysis, asymptotic *p*-values, exact *p*-values using 10,000 permutation results, and DA using 1000 *p*-values were calculated and corrected for multiple testing by imposing a false discovery rate (FDR) of 0.05. To assess the model adequacy, QQ-plots of original, exact and DA *p*-values were generated, and genomic inflation factors, *λ*_*gc*_, were calculated. This *λ*_*gc*_ is the ratio of the median of the observed test statistics divided by the expected median of an asymptotic distribution. If a *λ*_*gc*_ is greater than 1, this may suggest inflation in the test statistics and may indicate the presence of systemic bias such as hidden population structures, latent covariates, technical artifacts, etc. [32].

#### Analyzing HD data with simulated covariates

To evaluate the effect of covariates in a model, the same method for generating covariates in our simulation study was applied to the HD data set to create simulated covariates. In this real data application, we focused on a moderate covariate effect on HD status (CovOR = 1.2). The HD data were analyzed using the NB GLM in DESeq2 with Model A and using the FL regression with Model B with 1000 replicated sets of simulated covariates (*C* = 1, 2, 3, 5, or 10). The change of *λ*_*gc*_ with the addition of a varying number of simulated covariates in a model was evaluated.

## Results

### Simulation results of NB vs. Logistic regressions

#### Type-I error simulations

Type-I error rates from the simulated results of the scenarios at two alpha levels are presented in Table 2. All analyses presented in Table 2 were converged. The NB regressions using ML, QL and true dispersions show almost identical levels of performance (see Additional file 2: Table S1). When the sample size is small or the dispersion is high, NB regression shows inflated Type-I error rates but the CL and BL regressions are conservative (see Table 2). Large sample size and low dispersion generally yielded Type-I error rates that were close to the specified alpha levels as shown in Additional file 3: Figure S1. The increment of *μ*_*D=0*_ is not influential. The FL regression performs well or presents moderate conservativeness at both alpha levels. The Type-I error rates of the FL regression are less affected by the small sample size and the large dispersion than other logistic regressions. The Type-I error rates of additional scenarios exhibit patterns that are consistent with results in Table 2. The unbalanced design results shown in Additional file 4: Table S2 are consistent with the balanced design results in Table 2.

**Table 2:** Type-I error rates of regression methods from the balanced design having *μ*_*D=0*_ = 1000

Alpha	*N*_*D=1*_	Disp	NB	CL	BL	FL
0.05	10	0.01	0.066	0.023	0.023	0.044
	10	1	0.094	0.016	0.017	0.039
	25	0.01	0.057	0.044	0.040	0.049
	25	1	0.064	0.034	0.032	0.044
	75	0.01	0.054	0.050	0.048	0.051
	75	1	0.056	0.045	0.043	0.048
	500	0.01	0.049	0.049	0.048	0.049
	500	1	0.054	0.052	0.052	0.053
0.01	10	0.01	0.018	<0.001	<0.001	0.007
	10	1	0.032	<0.001	0.001	0.007
	25	0.01	0.014	0.005	0.004	0.011
	25	1	0.019	0.002	0.002	0.009
	75	0.01	0.012	0.009	0.008	0.010
	75	1	0.013	0.007	0.006	0.010
	500	0.01	0.011	0.010	0.010	0.011
	500	1	0.011	0.010	0.010	0.011

In most scenarios, the DA method reduces the inflation observed with NB regression and the controls conservativeness observed with the CL, BL, and FL regressions. However, with small sample size, conservative results are still observed at alpha level 0.01 for the CL and BL models. The DA method with NB and FL regressions showed well-controlled Type-I error rates at all alpha levels even with small sample size.

#### Power simulations

**Fig. 1:**
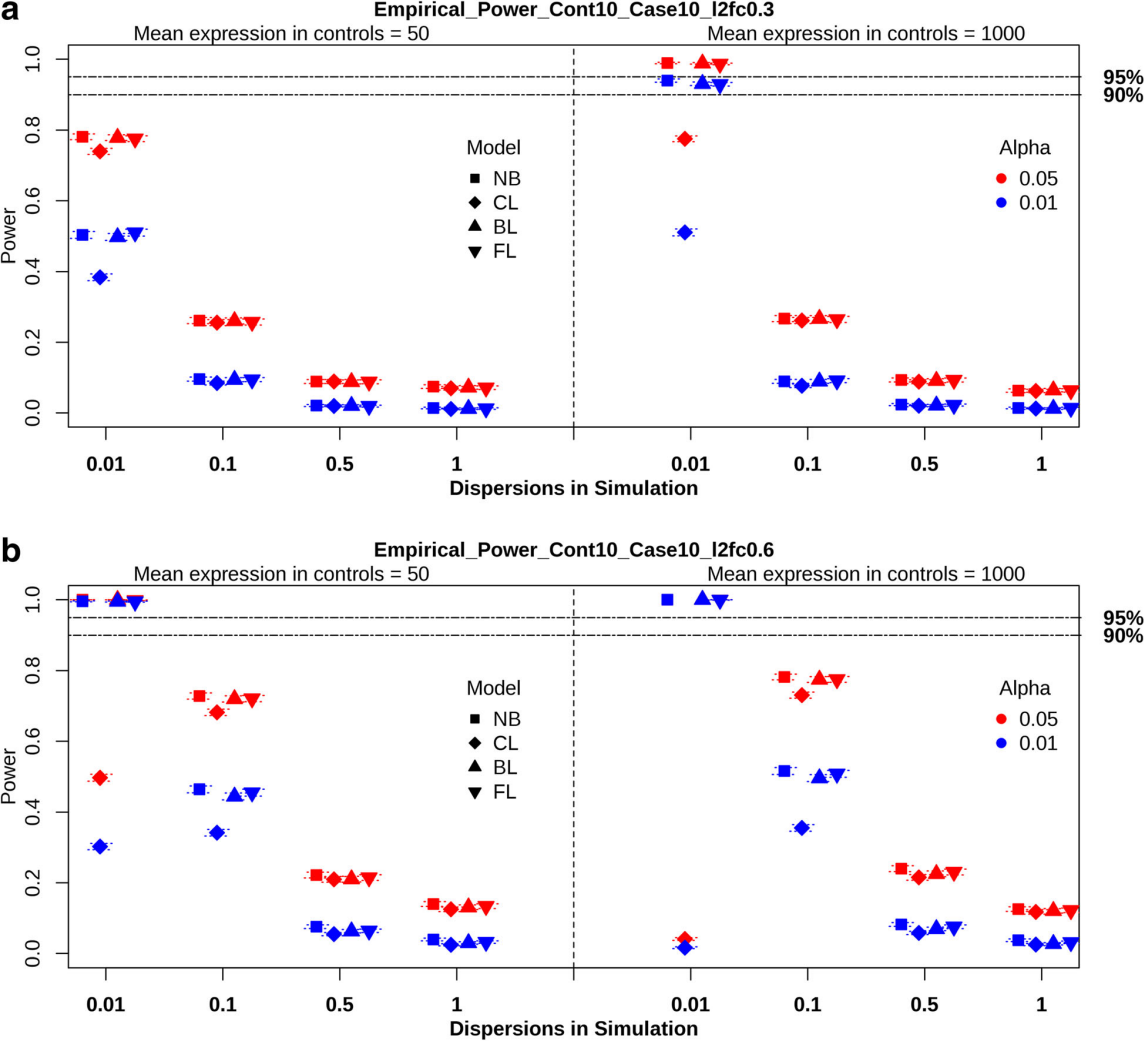
Empirical power of regression methods from the balanced design with *N*_*D=1*_ = 10. Power of the Negative Binomial with true dispersion (NB), Classic Logistic (CL), Bayes Logistic (BL), and Firth’s Logistic (FL) regressions at alpha levels of 0.05 and 0.01 are shown. The *black* dotted horizontal lines represent 95 and 90% power. Mean expression values (*μ* = 50 and 1000) are separated by *black* dotted vertical lines. Four dispersion values (0.01, 0.1, 0.5, and 1) are placed within each mean expression value. Dotted lines within each symbol imply 95% confidence interval. **a** The figure represents the results from the balanced design with *N*_*D=1*_ = 10 and log2fc = 0.3. **b** This figure shows the summary from the balanced design with *N*_*D=1*_ = 10 and log2fc = 0.6

We summarize the empirical power results from the balanced design with 10 cases in Fig. 1. The performance of the NB regressions with ML, QL and true dispersions are almost identical, as seen in Additional file 5: Table S3. Larger sample sizes increase power for all regression methods. The influence of mean expression in controls appears with small log2fc in Fig. 1. When sample size, log2fc, and dispersion are small, increase of mean expression in controls leads to an increase of power at both alpha levels. When log2fc is large and dispersion is small, the CL regression shows very low power. The NB, BL, and FL regressions gain power with large log2fc and low dispersion. These three regression methods have comparable empirical power and CL regression results in lower power in all scenarios.

### Application to RNA-Seq data in Huntington’s disease

#### Type-I error permutations

**Fig. 2:**
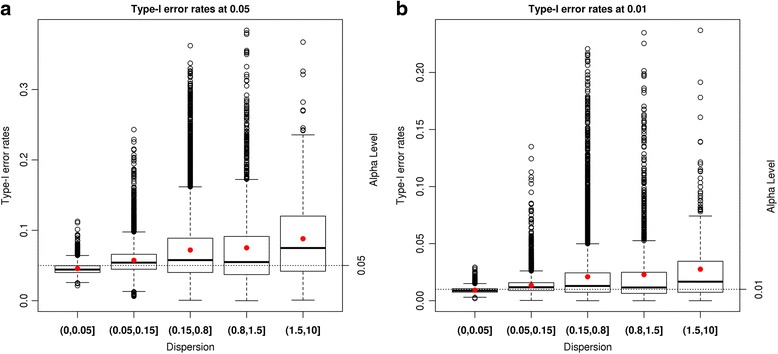
Type-I error rates from DESeq2 analysis of the permuted HD data. Type-I error rates from the DESeq2 (negative binomial model) analysis of the permuted HD data at alpha levels of 0.05 and 0.01 are presented in the figure. Each *black* empty dot represents the Type-I error rate of a gene. The *red* dots denote average values of Type-I error rates in each category of dispersion groups. The *black* dotted horizontal lines are our alpha levels. **a** shows Type-I error rates of genes having mean expression value of greater than 3 at alpha level of 0.05. **b** displays Type-I error rates of genes having mean expression value of greater than 3 at alpha level of 0.01

**Fig. 3:**
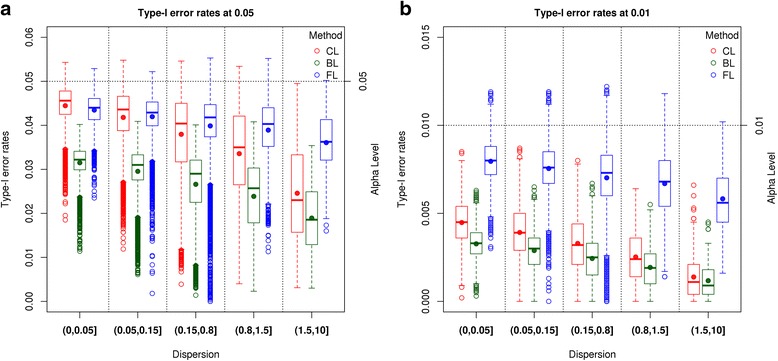
Type-I error rates from logistic regressions of the permuted HD data. Figure 3 contains Type-I error rates from Classical Logistic (CL), Bayes Logistic (BL), Firth’s Logistic (FL) regressions of the permuted HD data at alpha levels of 0.05 and 0.01. Each empty dot represents Type-I error rate of a gene. The dots filled with colors inside of boxes denote average values of Type-I error rates in each category of dispersion groups. The *black* dotted horizontal lines are our alpha levels. **a** shows Type-I error rates of genes having mean expression value of greater than 3 at alpha level of 0.05. **b** displays Type-I error rates of genes having mean expression value of greater than 3 at alpha level of 0.01

The Type-I error rates from the permuted data sets at two alpha levels are shown in Figs. 2 and 3. We categorize genes in 5 groups by the estimated dispersion of a gene: (0, 0.05), (0.05, 0.15), (0.15, 0.8), (0.8, 1.5), and (1.5, 10). We define genes with dispersion >0.8 as largely dispersed.

In the NB results from DESeq2, as dispersion increases, the Type-I error rates increase when genes are in the categories of (0, 0.05), (0.05, 0.15), and (0.15, 0.8). However, genes in the (0.8, 1.5), and (1.5, 10) categories exhibit decreasing Type-I error rates (Additional file 6: Figure S2). Genes in the (0.8, 1.5), and (1.5, 10) categories largely have very low mean expression values. After excluding genes having mean expression values less than 3, Type-I error rates increase as the estimated dispersion increases as shown in Fig. 2. These increasingly liberal Type-I error rates are observed at both alpha levels and are consistent with our simulation results.

In the CL, BL and FL regression results, we observe that genes in the categories of (0, 0.05), (0.05, 0.15), and (0.15, 0.8) produce increasingly conservative Type-I error rates at both alpha levels, as presented in Fig. 3. However, these increasingly conservative Type-I error rates are attenuated in the (0.8, 1.5), and (1.5, 10) categories (Additional file 7: Figure S3). Because we observe this inconsistent pattern of Type-I error rates among extremely lowly expressed genes in the DESeq2 results, we also examined the set of genes excluding those with mean expression values less than or equal to 3. After exclusion of genes with low expression, the remaining genes show consistent increasingly conservative Type-I error rates as dispersion increases as shown in Fig. 3. Although Type-I error rates from the FL regression are also more conservative when dispersion is large, Type-I error rates are relatively well controlled compared to CL and BL regressions. The Type-I error rates observed in the real data set using logistic regression confirm our simulation results.

The DA method controls Type-I error rates well for the DESeq2 results (Additional file 8: Figure S4) and the FL regression results (Additional file 9: Figure S5) at both alpha levels, regardless of dispersions of all genes. The Type-I error rates from CL and BL regressions at significance level of 0.01 are conservative as seen in Additional file 9: Figure S5.

We also observed the bias of the regression methods using permuted HD data sets. As shown in Additional file 10: Figure S6, FL regression revealed the smallest bias.

#### HD results from the NB vs. logistic regressions

We analyze the HD data using NB GLM in the DESeq2 R-package, and using CL, BL, and FL regressions with the R-functions described in the Methods section. All regression results are corrected with the DA method using 1000 permutation results, and are adjusted for multiple testing using an FDR of 0.05. The Q-Q plots and *λ*_*gc*_ are shown in Additional file 11: Figure S7. The DA method reduced the mean *λ*_*gc*_ from the results of DESeq2 and increased the mean *λ*_*gc*_ from the results of the CL, BL, and FL regressions. As shown in Additional file 12: Figure S8, we identified 3,203 genes that were significant across all methods. The FL regression also identified 307 genes as DE genes that were not identified by the other methods. The DESeq2 approach identified 944 genes that were not significant using the other methods. The 10 most significant genes (FDR < 0.05) from FL regression that are not significant (FDR > 0.05) in the DESeq2 but significant in CL, BL and FL regressions are shown in Table 3. The most significant gene is *SLC1A6* with *p*-value equal to 3.2E-06 from FL regression. Of the genes that are not significant (FDR > 0.05) in the CL, BL and FL analyses, the 10 most significant (FDR < 0.05) from DESeq2 are shown in Additional file 13: Table S4. The genes that are significant in FL analysis are listed in Additional file 14: Table S5.

**Table 3:** Top 10 significant genes from FL regression among genes not significant in DESeq2

Gene	Mean. Exp. Case	Mean. Exp. Cont	Disp	NB.Pval	CL.Pval	BL.Pval	FL.Pval
*SLC1A6*	374	554	0.29	0.039	4.3E-04	4.5E-04	3.2E-06
*SERHL2*	210	163	0.17	0.016	3.2E-04	6.3E-03	1.2E-05
*KCNK9*	315	454	0.30	0.063	3.0E-04	9.2E-04	1.7E-05
*DISP2*	687	937	0.21	0.047	5.5E-04	8.2E-04	4.3E-05
*SPOCK2*	12370	15649	0.09	0.010	8.9E-04	1.1E-03	8.0E-05
*C20orf27*	726	934	0.11	0.019	5.9E-04	2.4E-04	9.5E-05
*IST1*	3388	3134	0.02	0.009	5.7E-04	4.5E-03	9.6E-05
*ARC*	596	1058	0.40	0.030	1.1E-03	1.2E-03	1.0E-04
*STRADB*	980	844	0.03	0.013	1.4E-03	1.5E-03	1.0E-04
*PCP4*	734	1330	0.37	0.086	1.1E-03	2.2E-03	1.2E-04

### Simulation results of various covariate models from the NB and FL regressions

The properties of the NB regression that are shown in *Simulation results of NB vs. Logistic regressions* are also presented in the simulation results with various covariate models.

#### Type-I error simulations

Type-I error rates from the simulated data to evaluate the inclusion of covariates in the models are presented in Table 4. When dispersion is small, an increasing number of NP covariates do not increase Type-I error rates in the NB models. Although the number of covariates appears to increase Type-I error rates when dispersion is 0.01 and CovOR is 5 (Table 4), this slightly increased Type-I error rate is close to the Type-I error rates in the model when dispersion is 0.01 and CovOR is 1.2. Adding more NP covariates when the dispersion is large increases Type-I error rates. The effects of large CovOR on Type-I error rates are not notable in NB models. Large sample size weakens the inflation that arises from a large number of NP covariates within large dispersion in the NB model. Type-I error rates with distinct dispersions are almost identical at both alpha levels (Additional file 15: Table S6).

**Table 4:** Type-I error rates with covariate models from balanced design of *N*_*D=1*_ = 10 and *μ*_*D=0*_ = 1000

Disp	CovOR	Ncov	Alpha = 0.05		Alpha = 0.01	
			NB	FL	NB	FL
0.01	1.2	1	0.071	0.049	0.023	0.01
0.01	1.2	5	0.076	0.05	0.026	0.009
0.01	5	1	0.061	0.041	0.018	0.006
0.01	5	5	0.08	0.021	0.024	0.001
1	1.2	1	0.103	0.041	0.036	0.006
1	1.2	5	0.151	0.045	0.067	0.008
1	5	1	0.102	0.039	0.037	0.007
1	5	5	0.142	0.02	0.061	0.001

The same covariates that are non-predictive (NP) covariates in a NB model are non-confounding predictive (NCP) covariates in logistic models. Unlike the NB regression, even with small sample size (Table 4), when the CovOR is small, the FL regression is robust regarding the increment in the number of NCP covariates. When CovOR is large, Type-I error rates from FL regression become very conservative as the number of NCP covariates increase. Type-I error rates are not affected by large dispersion. When sample size is increased, Type-I error rates are less affected by increased number of NCP covariates with large CovOR.

In all scenarios, the DA method controls Type-I error rates well in the NB and FL regressions at both alpha levels. The newly approximated distribution of test statistics diminishes deviated Type-I error rates that were not controlled when many covariates were included in the NB and FL models.

In addition to Type-I error rates, the bias is also calculated for each scenario. As shown in Additional file 16: Table S7, as dispersion increases, bias from NB regression increases. However, the bias from FL regression is robust with increasing dispersion. The bias does not change much for any of the models, as CovOR and the number of covariates changes.

#### Power simulations

**Fig. 4:**
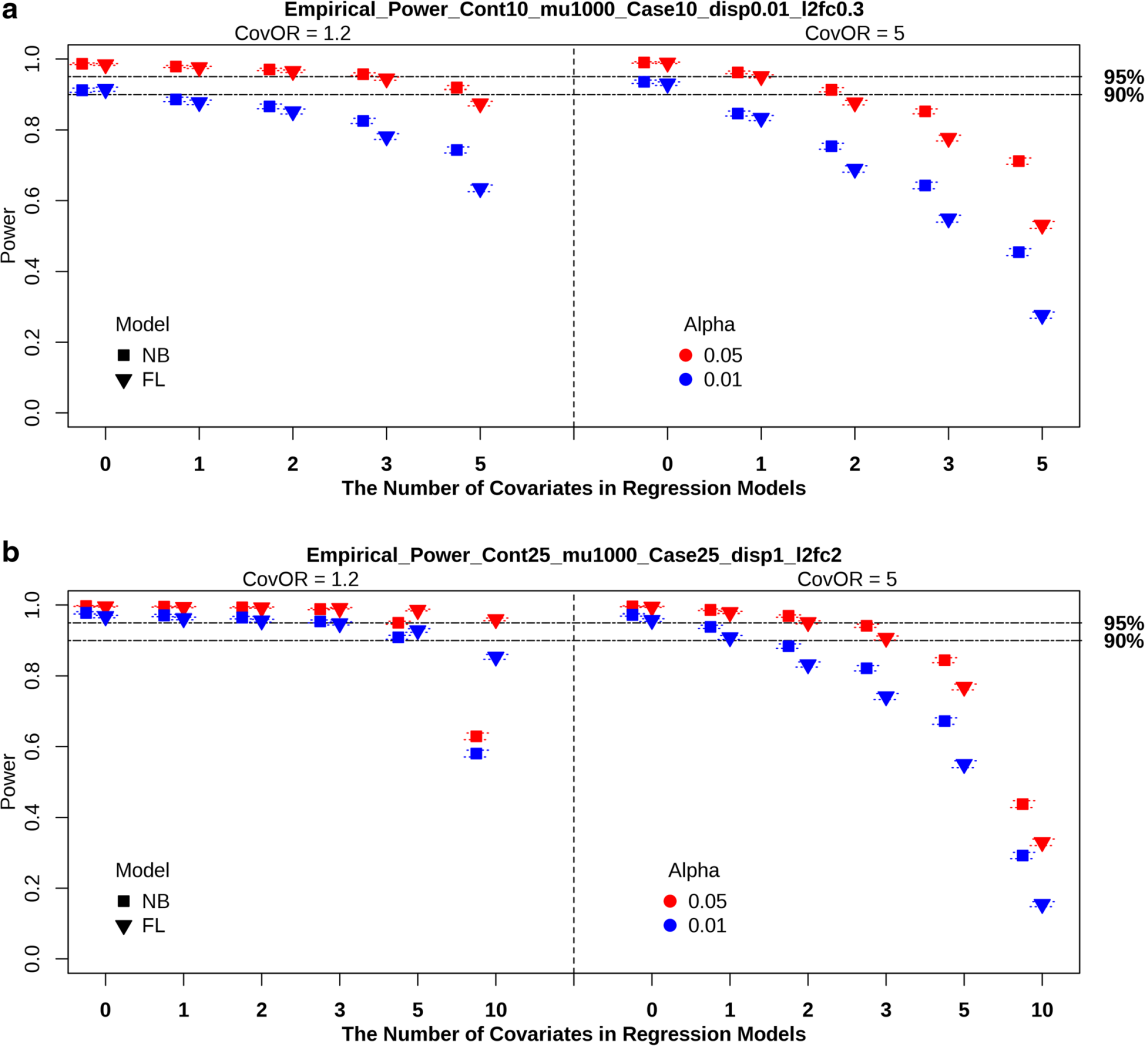
Empirical power of covariate models from balanced design with *N*_*D=1*_ = 10 and *μ*_*D=0*_ = 1000. The power of Negative Binomial with true dispersion (NB), and Firth’s Logistic (FL) regressions at significance level 0.05 and 0.01 is shown in the figure. *Black* dotted horizontal lines represent 95 and 90% power. The odds ratios between covariates and case–control status (CovOR = 1.2 and 5) are partitioned by vertical *black* dotted lines. The number covariates (0, 1, 2, 3, 5 (, and 10)) in the model are positioned within each CovOR. Dotted lines within each symbol represent the 95% confidence interval. **a** Balanced design from *N*_*D=1*_ *= 10*, *μ*_*D=0*_ = 1000, dispersion = 0.01, and log2fc = 0.3. **b** Balanced design of *N*_*D=1*_ *= 25*, *μ*_*D=0*_ = 1000, dispersion = 1, and log2fc = 2

The results of the power simulations to evaluate the inclusion of covariates in the models are summarized in Fig. 4. The empirical power of the NB regressions using different dispersion estimation methods was similar for all power scenarios (Additional file 17: Table S8). When sample size is increased the overall power is increased in both NB and FL regression.

In our simulation, the power of NB and FL regression is affected by three factors 1) Dispersion, 2) CovOR, and 3) Number of NP/NCP covariates in a model, when sample size is fixed. Large dispersion, large CovOR, and increasing number of NP/NCP covariates in a model decrease power. NB regression is less sensitive to an increase of NP covariates with small dispersion. As shown Fig. 4a, NB regression results in marginally more power than FL regression when the number of covariates is large and dispersion is small. When dispersion is large but CovOR is small, the loss of power in NB regression is more sensitive to the increase in number of NP covariates than in FL regression as seen in Fig. 4b. Especially, in Fig. 4b with the CovOR of 1.2, the power of FL regression with 10 covariates in a model is more powerful than NB regression with 10 covariates. Regardless of dispersion, when CovOR and the number of covariates in a model are large, NB regression shows slightly better power than FL regression. This is demonstrated in Fig. 4 with CovOR equal to 5.

#### HD results from covariate models

The HD data were analyzed using DESeq2 and FL regression with additional simulated covariates. A summary of *λ*_*gc*_ is presented in Table 5. An increase of the NP/NCP covariates leads to a marginally lower median of the *λ*_*gc*_. The standard deviations of the *λ*_*gc*_ are increased with the increase of NP/NCP covariates.

**Table 5:** Summary of *λ*_*gc*_ from HD analyses with simulated covariates

Ncov	Median_NB	SD_NB	Median_FL	SD_FL
1	4.05	0.10	3.50	0.16
2	4.02	0.14	3.46	0.22
3	4.00	0.17	3.40	0.27
5	3.93	0.22	3.28	0.35
10	3.74	0.29	2.95	0.53

## Discussion

In this study, we propose using a logistic regression framework as an alternative to NB regression to analyze RNA-Seq data for case–control studies. We have shown in our simulations that FL regression performs well in terms of controlling Type-I error rates and shows comparable empirical power. The dispersion is not estimated in the logistic framework, thus avoiding potential false association resulting from incorrectly estimated dispersion. The simulations presented focused on single genes varying relevant parameters (mean, dispersion, log_2_fold-change); transcriptome-wide data were not simulated.

The Type-I error simulations presented demonstrate that NB regression has inflated Type-I error rates with small sample size. The degree of inflation is varied by the scale of the dispersion parameter with constant sample size. The relationship between increased Type-I error and dispersion was confirmed through permutation of a real data set. Although large sample size reduced the inflation from NB, the high cost of RNA-Seq technology and difficulty of obtaining certain sample tissues may preclude a larger sample size in some studies. The distinct Type-I error rates observed with varying dispersion parameter values may violate the general assumption that *p*-values from non-DE genes follow a uniform distribution. However, the current simulation and permutation studies validate that the DA method is a suitable alternative approach that controls Type-I error rates in all regression methods.

The empirical power of the NB, BL, and FL regressions are comparable across all scenarios. Lower power was observed for CL regression, which appears to be driven by scenarios of complete separation and a failure to converge. With large log2fc and small dispersion, simulated data are likely to show complete separation; in these scenarios the NB, BL and FL regressions are more powerful. For many circumstances with small sample size, the CL regression demonstrated the lowest empirical power among all methods because the CL regression is not able to accommodate complete separation.

Analysis of the HD data showed *λ*_*gc*_ was decreased after applying the DA method to the results from NB GLM in DESeq2 but was increased after applying DA method to the results from CL, BL and FL regression. The exact *p*-values from 10,000 permutations revealed the same pattern. This pattern is consistent with our simulation results where Type-I error rates were inflated in the NB framework and conservative in the logistic framework when test statistics were compared with a theoretical asymptotic distribution.

Although it is unknown which genes are truly DE in the HD data set, we compared DE genes identified in the HD data by different statistical approaches. We found that *SLC1A6* (solute carrier family 1, member 6; *EAAT4*) did not show evidence of association with HD when using DESeq2, but was highly significant when using FL regression, as shown in Table 3. *SLC1A6*, which is highly expressed in the cerebellum of human brain compared to other regions [33], showed lower levels of expression in prior studies of mood disorder diseases such as bipolar and major depression disorders in the striatum in situ hybridization study [34]. In addition, Utal et al. [35] showed that Purkinje cell protein 4 (*PCP4*), also known as *PEP-19*, had dramatic reduction in HD. This gene was not significantly associated with HD status when using DESeq2 (*p*-value = 0.086) but showed strong association when using FL regression (*p*-value = 1.2 × 10^−4^). We also found that some genes expressed highly in both cases and controls may not be detected in the NB framework, because it utilizes the ratio of mean expressions of cases and controls. For instance, the normalized mean expression value of *SPOCK2* is 12,370 in cases and 15,649 in controls. Although the difference of the means is large, the gene might not be statistically significant due to the small effect size (log2fold-change = −0.34) in the NB framework. However, this gene is strongly associated with HD in our logistic framework (Table 3). *SPOCK2*, also known as *SPARC*/osteonectin and Testican-2, plays an important role in the central nervous system [36]. As a member of the testican group, the expression in various neuronal cell types including cell types cerebral cortex, thalamus, hippocampus, cerebellum was reported by in situ hybridization [36]. Several studies showed evidence of associations with prostate, colon, and breast cancer and bronchopulmonary dysplasia [37, 38].

The top genes that showed associations exclusively in NB GLM, except for gene *S100A11*, have low average counts as shown in Additional file 13: Table S4. The estimated dispersions for these genes are also fairly large suggesting that they may be false positives.

The effect of including covariates has not been investigated for RNA-Seq studies. Identifying relationships with covariates for all genes is computationally demanding and existing software do not allow for defining gene-wise models for all genes, which makes this approach challenging. Therefore, RNA-Seq studies that include covariates in a single model applied to all genes will likely result in some gene expression models that include unassociated covariates. Hence, it is important to investigate the effect of NP covariates in RNA-Seq analysis.

Simulations that included NP covariates in the NB model showed inflated Type-I error rates and a loss of power. With large dispersion, this inflation and loss of power becomes severe. The Type-I error in the FL regression is not notably affected by the increment of number of NCP covariates when the CovOR is small. With large CovOR and increased number of NCP covariates, conservative Type-I error rates are observed. The DA method effectively controls the increase of Type-I error rates even with larger CovOR and high number of NP/NCP covariates. Our empirical power results show that the FL regression is more greatly influenced by the increase of covariates than the NB regression, when CovOR is large.

Our HD analyses with simulated NP/NCP covariates demonstrated that an increase in the number of NP/NCP covariates results in a less stable *λ*_*gc*_(Table 5). Adding more NP covariates in an NB model slightly decreases the median of *λ*_*gc*_, and hence an increased in the median of *p*-values. In other words, many *p*-values in a set are generally increased. Based on our simulation results with NP covariates, Type-I error rates of largely dispersed genes are likely to be inflated and power of differentially expressed genes are likely to be decreased. Therefore, this slightly decreased median may indicate that the loss of power is greater than the gain of Type-I error rates.

Adding more NCP covariates in a model also slightly decreases the median of the *λ*_*gc*_ in FL regression. This decreased median might be caused by the loss of power, and this loss may occur from NCP covariates in a model according to our simulation results. Under a moderate CovOR, the number of NCP covariates in a model does not affect the Type-I error rates.

The change in the median *λ*_*gc*_ with additional covariates is larger in FL regression than in NB regression because the FL regression results are solely affected by the loss of power. NB regression results are influenced by both increased Type-I error and decreased power. The standard deviation of *λ*_*gc*_ is increased with adding NP/NCP covariates in a model. This means that the results generated from a model that includes many covariates is not likely to be reliable, even if these covariates are associated with case–control status but not gene expression.

## Conclusions

In conclusion, unlike NB, CL and BL regressions, FL regression controls Type-I error rates well and maintains comparable power even with small sample size. Firth’s logistic regression is an excellent alternative to NB regression for analysis of RNA-Seq data in case–control studies. We recommend implementing the DA method in analysis of RNA-Seq data to appropriately control Type-I error rates. If computational burden of permutations required for the DA method precludes using this approach, FL regression is the best option for controlling Type-I errors with comparable power to NB regression. However, a parsimonious model is necessary to obtain robust results in the FL regression setting. This approach can be extended in multiple classes of disease status using a multinomial logistic regression method.

## Additional files

Additional file 1: Supplementary Method. This document provides detailed descriptions of the dispersion estimation method, Type-I error rates and empirical power calculations, procedures of data adaptive method using cross-validation technique, and Huntington’s disease data. (DOCX 56 kb)
Additional file 2: Table S1. Type-I error rates of the NB regression from the balanced design with *N*_*D=1*_ = 10. Mean: The mean expression values in cases and controls, Disp: Dispersion, NB: Negative Binomial regression, MLD: Maximum likelihood estimated dispersion, QLD: Quasi-likelihood estimated dispersion, TD: True dispersion specified in the simulation. (DOCX 55 kb)
Additional file 3: Figure S1. Type-I error rates of regression methods from the balanced design. Type-I error rates of the Negative Binomial with true dispersion (NB), Classic Logistic (CL), Bayes Logistic (BL), and Firth’s Logistic (FL) regressions at alpha levels of 0.05 and 0.01 are shown. The black dotted horizontal lines represent 5 and 1% of Type-I error rates. Dispersion values (*ϕ* = 0.01 and 1) are separated by black dotted vertical lines. Four values of the number of cases (10, 25, 75 and 500) are placed within each dispersion value. Dotted lines within each symbol imply 95% confidence interval. Figure S1 (A): The figure presents the Type-I error rates when *μ =* 50. Figure S1 (B): This figure shows the Type-I error rates when *μ =* 1000. (PNG 399 kb)
Additional file 4: Table S2. Type-I error rates of regression methods from the unbalanced design with *μ*_*D=0*_ = 1000. Alpha: Significance levels, *N*_*D=1*_: The number of cases, *N*_*D=0*_: The number of controls, Disp: Dispersion, NB: Negative binomial regression with true dispersion, CL: Classical logistic regression, BL: Bayes logistic regression, FL: Firth’s logistic regression. (DOCX 65 kb)
Additional file 5: Table S3. Empirical power of NB regression from the balanced design with *N*_*D=1*_ = 10 and log2fc = 0.3. Mean: The mean expression values in cases and controls, Disp: Dispersion, NB: Negative Binomial regression, MLD: Maximum likelihood estimated dispersion, QLD: Quasi-likelihood estimated dispersion, TD: True dispersion specified in the simulation. (DOCX 56 kb)
Additional file 6: Figure S2. Type-I error rates from DESeq2 analysis of the permuted HD data. This contains Type-I error rates from DESeq2 (negative binomial model) analysis of the permuted HD data at alpha levels of 0.05 and 0.01. Each black empty dot represents Type-I error rate of a gene. The red dots denote average values of Type-I error rates in each category of dispersion groups. The black dotted horizontal lines are our alpha levels. Figure S2 (A) shows Type-I error rates of all genes at alpha level of 0.05. Figure S2 (B) displays Type-I error rates of all genes at alpha level of 0.01. (PNG 309 kb)
Additional file 7: Figure S3. Type-I error rates from logistic model analyses of the permuted HD data. Figure S3 contains Type-I error rates from Classical Logistic (CL), Bayes Logistic (BL), Firth’s Logistic (FL) regressions of the permuted HD data at alpha levels of 0.05 and 0.01. Each empty dot represents Type-I error rate of a gene. The dots filled with colors inside of boxes denote average values of Type-I error rates in each category of dispersion groups. The black dotted horizontal lines are our alpha levels. Figure S3 (A) shows Type-I error rates of all genes at alpha level of 0.05. Figure S3 (B) displays Type-I error rates of all genes at alpha level of 0.01. (PNG 470 kb)
Additional file 8: Figure S4. Type-I error rates from DESeq2 analysis with the DA method using the permuted HD data. Figure S4 contains Type-I error rates from DESeq2 (negative binomial model) analysis with DA method of the permuted HD data at alpha levels of 0.05 and 0.01. Each black empty dot represents Type-I error rate of a gene. The red dots denote average values of Type-I error rates in each category of dispersion groups. The black dotted horizontal lines are our alpha levels. Figure S4 (A) summarizes Type-I error rates of all genes with DA method at alpha level of 0.05. Figure S4 (B) displays Type-I error rates of all genes with DA method at alpha level of 0.01. (PNG 223 kb)
Additional file 9: Figure S5. Type-I error rates from logistic model analyses with the DA method using the permuted HD data. Figure S5 presents Type-I error rates from Classical Logistic (CL), Bayes Logistic (BL), Firth’s Logistic (FL) regressions with the DA method of the permuted HD data at alpha levels of 0.05 and 0.01. Each empty dot represents Type-I error rate of a gene. The dots filled with colors inside of boxes denote average values of Type-I error rates in each category of dispersion groups. The black dotted horizontal lines are our alpha levels. Figure S5 (A) shows Type-I error rates of all genes with DA method at alpha level of 0.05. Figure S5 (B) represents Type-I error rates of all genes with DA method at alpha level of 0.01. (PNG 453 kb)
Additional file 10: Figure S6. Bias from regression methods using the permuted HD data with *μ*_*g*_ > 3. Figure S6 contains bias from Negative Binomial regression using DESeq2, Classical Logistic regression (CL), Bayes Logistic regression (BL), and Firth’s Logistic regression (FL). Each black empty dot represents the bias of a gene. The black dotted horizontal line is no bias point. The bias of each gene is calculated using effect sizes of 10,000 permutations. (PNG 53 kb)
Additional file 11: Figure S7. Q-Q plots of the HD Analyses. Figure S7 exhibits the Q-Q plots from the HD analysis adjusting for age at death and RIN from DESeq2 (A), and Classical (B), Bayes (C), and Firth’s (D) Logistic regressions. Each regression method contains three different ways of calculating *p*-values (Original, DA, and Perm). “Original” *p*-values (Blue dots) are estimated from asymptotic distribution. “DA” *p*-values (Black dots) are evaluated from data adaptive asymptotic distribution using 1,000 permutations. “Perm” *p*-values (Yellow dots) are calculated using 10,000 permutations. (PNG 157 kb)
Additional file 12: Figure S8. Venn diagram of HD analysis results using DA method. Each colored circle represents a different regression method. The numbers inside of the circles are the number of genes significant at FDR 0.05 based on *p*-values adjusted using the Data Adaptive (DA) method. There were 3,203 significant genes in common across all the methods. The FL identified the largest number of significant genes compared to CL and BL. The NB independently identified 944 genes. (PNG 474 kb)
Additional file 13: Table S4. Top 10 significant genes from DESeq2 among genes not significant in logistic regressions. Mean.Exp.Case: Normalized mean expression value in cases, Mean.Exp.Cont: Normalized mean expression value in controls, Disp: Dispersion, NB.Pval: *P*-values from negative binomial regression with true dispersion, CL.Pval: *P*-values from classical logistic regression, BL.Pval: *P*-values from Bayes logistic regression, FL.Pval: *P*-values from Firth’s logistic regression. (DOCX 53 kb)
Additional file 14: Table S5. All significant genes in FL regressions using the DA method. Mean.Exp.Case: Normalized mean expression value in cases, Mean.Exp.Cont: Normalized mean expression value in controls, Disp: Dispersion, NB, CL, BL, FL: *P*-values from negative binomial regression, classical logistic regression, Bayes logistic regression, Firth’s logistic regression. (XLS 1013 kb)
Additional file 15: Table S6. Type-I error rates of the NB regression from the balanced design with *N*_*D=1*_ = 10 and *μ* = 1000. Disp: Dispersion, CovOR: Odds ratios between covariates and case–control status, Ncov: The number of covariates in a model, NB: Negative binomial regression, MLD: Maximum likelihood estimated Dispersion, QLD: Quasi-likelihood estimated Dispersion, TD: The dispersion is used for the sampling. (DOCX 59 kb)
Additional file 16: Table S7. Bias with covariate models from the balanced design of *N*_*D=1*_ = 10 and *μ*_*D=0*_ = 1000. Disp: Dispersion, CovOR: Odds ratios between covariates and case–control status, Ncov: The number of covariates in a model, NB_TD: Negative binomial regression with the dispersion is used for the sampling, FL: Firth’s logistic regression. (DOCX 47 kb)
Additional file 17: Table S8. Type-I error rates of the NB regression from the balanced design with *N*_*D=1*_ = 10, *μ*_*D=0*_ = 1000, and log2fc = 0.3. Disp: Dispersion, CovOR: Odds ratios between covariates and case–control status, Ncov: The number of covariates in a model, NB: Negative binomial regression, MLD: Maximum likelihood estimated Dispersion, QLD: Quasi-likelihood estimated Dispersion, TD: The dispersion is used for the sampling. (DOCX 59 kb)
Additional file 18: R code. This R code regenerates the simulated data sets. (R 6 kb)

## Abbreviations

AAD: Age at death
BL: Bayes logistic
CL: Classical logistic
CovOR: Covariate OR
DA: Data adaptive
DE: Differentially expressed
FDR: False discovery rate
FL: Firth’s logistic
GLM: Generalized linear model
HD: Huntington’s disease
log2fc: log_2_ fold-change
ML: Maximum-likelihood
NB: Negative Binomial
NCP: Non-confounding predictive
NGS: Next generation sequencing
NP: Non-predictive
QL: Quasi-likelihood
RIN: RNA Integrity number
RNA: Ribonucleic acid
RNA-Seq: RNA sequencing

## References

[1] Dillies M-A, Rau A, Aubert J, Hennequet-Antier C, Jeanmougin M, Servant N (2013). A comprehensive evaluation of normalization methods for Illumina high-throughput RNA sequencing data analysis. Brief Bioinform.

[2] Anders S, Huber W (2010). Differential expression analysis for sequence count data. Genome Biol.

[3] Love MI, Huber W, Anders S (2014). Moderated estimation of fold change and dispersion for RNA-seq data with DESeq2. Genome Biol.

[4] Robinson MD, McCarthy DJ, Smyth GK (2010). edger: a bioconductor package for differential expression analysis of digital gene expression data. Bioinformatics.

[5] Seyednasrollah F, Laiho A, Elo LL. Comparison of software packages for detecting differential expression in RNA-seq studies. Brief Bioinform. 2013;16:59-70 10.1093/bib/bbt086 PMC4293378 24300110

[6] Tang M, Sun J, Shimizu K, Kadota K (2015). Evaluation of methods for differential expression analysis on multi-group RNA-seq count data. BMC Bioinformatics.

[7] Rapaport F, Khanin R, Liang Y, Pirun M, Krek A, Zumbo P (2013). Comprehensive evaluation of differential gene expression analysis methods for RNA-seq data. Genome Biol.

[8] Landau WM, Liu P (2013). Dispersion estimation and its effect on test performance in RNA-seq data analysis: a simulation-based comparison of methods. Chen L, editor. PLoS One.

[9] Soneson C, Delorenzi M (2013). A comparison of methods for differential expression analysis of RNA-seq data. BMC Bioinformatics.

[10] Hardcastle TJ, Kelly KA (2010). baySeq: empirical Bayesian methods for identifying differential expression in sequence count data. BMC Bioinformatics.

[11] Di Y, Schafer DW, Cumbie JS, Chang JH. The NBP Negative Binomial Model for Assessing Differential Gene Expression from RNA-Seq. Stat Appl Genet Mol Biol. 2011;10:1–28.

[12] Auer PL, Doerge RW. A Two-Stage Poisson Model for Testing RNA-Seq Data. Stat Appl Genet Mol Biol. 2011;10:1.

[13] Leng N, Dawson JA, Thomson JA, Ruotti V, Rissman AI, Smits BMG (2013). EBSeq: an empirical Bayes hierarchical model for inference in RNA-seq experiments. Bioinformatics.

[14] Tarazona S, Garcia-Alcalde F, Dopazo J, Ferrer A, Conesa A (2011). Differential expression in RNA-seq: a matter of depth. Genome Res.

[15] Li J, Tibshirani R (2013). Finding consistent patterns: a nonparametric approach for identifying differential expression in RNA-Seq data. Stat Methods Med Res.

[16] Van De Wiel MA, Leday GGR, Pardo L, Rue H, Van Der Vaart AW, Van Wieringen WN (2013). Bayesian analysis of RNA sequencing data by estimating multiple shrinkage priors. Biostatistics.

[17] Smyth GK (2004). Linear models and empirical Bayes methods for assessing differential expression in microarray experiments. Stat Appl Genet Mol Biol.

[18] Seyednasrollah F, Laiho A, Elo LL (2015). Comparison of software packages for detecting differential expression in RNA-seq studies. Brief Bioinform.

[19] Trapnell C, Hendrickson DG, Sauvageau M, Goff L, Rinn JL, Pachter L (2012). Differential analysis of gene regulation at transcript resolution with RNA-seq. Nat Biotechnol.

[20] Li J, Witten DM, Johnstone IM, Tibshirani R (2012). Normalization, testing, and false discovery rate estimation for RNA-sequencing data. Biostatistics.

[21] Sun J, Nishiyama T, Shimizu K, Kadota K (2013). TCC: an R package for comparing tag count data with robust normalization strategies. BMC Bioinformatics.

[22] Law CWC, Chen Y, Shi W, Smyth GGK (2014). Voom: precision weights unlock linear model analysis tools for RNA-seq read counts. Genome Biol.

[23] Yu L, Chibnik LB, Srivastava GP, Pochet N, Yang J, Xu J (2015). Association of brain DNA methylation in SORL1, ABCA7, HLA-DRB5, SLC24A4, and BIN1 with pathological diagnosis of alzheimer disease. JAMA Neurol.

[24] Bennett DA, Yu L, De Jager PL (2014). Building a pipeline to discover and validate novel therapeutic targets and lead compounds for alzheimer’s disease. Biochem Pharmacol.

[25] Labadorf A, Hoss AG, Lagomarsino V, Latourelle JC, Hadzi TC, Bregu J (2015). RNA sequence analysis of human huntington disease brain reveals an extensive increase in inflammatory and developmental gene expression. Ariga H, editor. PLoS One.

[26] McCullagh P, Nelder JA (1989). Generalized linear models. Second.

[27] Gelman A, Jakulin A, Pittau MG, Su Y-S (2008). A weakly informative default prior distribution for logistic and other regression models. Ann Appl Stat.

[28] Firth D (1993). Bias reduction of maximum likelihood estimates. Biometrika.

[29] Heinze G, Schemper M (2002). A solution to the problem of separation in logistic regression. Stat Med.

[30] Han F, Pan W (2010). A data-adaptive sum test for disease association with multiple common or rare variants. Hum Hered.

[31] Phipson B, Smyth GK (2010). Permutation P-values should never be zero: calculating exact P-values when permutations Are randomly drawn. Stat Appl Genet Mol Biol.

[32] Devlin B, Roeder K (1999). Genomic control for association studies. Biometrics.

[33] Furuta A, Martin L, Lin C-L, Dykes-Hoberg M, Rothstein JD (1997). Cellular and synaptic localization of the neuronal glutamate transporters excitatory amino acid transporter 3 and 4. Neuroscience.

[34] McCullumsmith R (2002). Striatal excitatory amino acid transporter transcript expression in schizophrenia, bipolar disorder, and major depressive disorder. Neuropsychopharmacology.

[35] Utal A, Stopka A, Roy M, Coleman P (1998). PEP-19 immunohistochemistry defines the basal ganglia and associated structures in the adult human brain, and is dramatically reduced in Huntington’s disease. Neuroscience.

[36] Vannahme C, Schübel S, Herud M, Gösling S, Hülsmann H, Paulsson M (2002). Molecular cloning of testican-2. J Neurochem.

[37] Hadchouel A, Durrmeyer X, Bouzigon E, Incitti R, Huusko J, Jarreau P-H (2011). Identification of SPOCK2 as a susceptibility gene for bronchopulmonary dysplasia. Am J Respir Crit Care Med.

[38] Chung W, Kwabi-Addo B, Ittmann M, Jelinek J, Shen L, Yu Y (2008). Identification of novel tumor markers in prostate, colon and breast cancer by unbiased methylation profiling. PLoS One.

