# Supplementary material for: Evaluation of logistic regression models and effect of covariates for case–control study in RNA-Seq analysis
Source: BMC Bioinformatics. 2017 Feb 6;18:91. doi: 10.1186/s12859-017-1498-y (PMC5294900; doi:10.1186/s12859-017-1498-y)
Supplement: Additional file 1: Supplementary Method. — This document provides detailed descriptions of the dispersion estimation method, Type-I error rates and empirical power calculations, procedures of data adaptive method using cross-validation technique, and Huntington’s disease data. (DOCX 56 kb) [file 12859_2017_1498_MOESM1_ESM.docx]

# Dispersion estimation methods

Implementation of NB regression requires the estimation of the dispersion parameter. Although several methods implemented in the RNA-Seq setting utilize data from across all genes to improve estimation we did not utilize those methods in our gene-focused simulations. NB regression approaches were implemented using either the true parameter value used in simulation or an ML or QL approach to estimating the dispersion parameter. The estimation of dispersion parameter was performed based on Model SA.

where *D* is case-control status and *Y* is gene expression.

## Maximum-likelihood (ML) estimated dispersion

ML estimates both the linear combination of unknown coefficients and the dispersion parameter (*φ*) in Model SA. We obtain by maximizing and this GLM is expedited by using iterative reweighted least square (IWLS) with a log link function[1].

An alternating iteration process was employed until both and were converged. The was estimated using score and information iterations in the *glm.nb* function of R-package “MASS” [2].

## Quasi-likelihood (QL) estimated dispersion

Because the ML estimated dispersion parameter is commonly underestimated an alternative estimation approach, the Quasi-likelihood model, was suggested[3]. This method estimates a dispersion parameter using a deviance statistic[4].

The ML estimate (whereis i^th^ row of the design matrix) *µ* was achieved by maximizing the NB log-likelihood given and *y_ig_*. This method is implemented in R-package “AMAP.seq” [5].

# Type-I error rates and empirical power

, (2.1)

(2.2)

(2.3)

where *m* is the number of simulations, *m^*^* is the number of converged simulations, and Q is alpha × *m^*^*

# Procedures of data adaptive method using cross-validation technique

1. The results from each Type-I error scenario were randomly and evenly partitioned into 10 groups.
2. Of the 10 groups, 9 were assigned as the training set (9000) and the remaining one was assigned as the testing (1000) set. Then, the scale (*a*) and location (*b*) parameters were estimated from test statistics using the training set.

*χ_g_* ~ *a*_g_ *χ*_1_+ *b_g_* where *χ_g_* is a test statistic of scenario *g* (3.1)

1. The p-values were re-generated using an adjusted chi-square distribution.
2. For all 10 combinations of testing and training set partitions, we performed steps (2) - (4), and Type-I error rates were re-calculated for Type-I error scenarios.

# Huntington’s disease (HD) data

The RNA was extracted from frozen brain tissue in prefrontal cortex Brodmann Area 9 from 20 HD cases and 49 controls who were neurologically normal at death and sequenced using Illumina HiSeq2000 technology for 100 nucleotide paired-end reads. These reads were aligned to the human reference genome (hg19) and annotated with Gencode database (v17). Only genes that have non-zero counts in more than half of the samples were kept for analysis, and extreme outliers in the raw counts were trimmed. After filtering, there were 28,087 genes in the final data set. Age at death (AAD) categorized into 4 groups and the RNA Integrity Number (RIN) defined as a binary variable specifying RIN > 7 or <= 7 were included in the model as covariates to prevent spurious associations. Because AAD was considered a non-ordinal, categorical variable, the total number of covariates is four in this model. The outlier correcting method implemented in DESeq2 was not applied because the outliers were already trimmed in the raw data.

# References

1. Lawless JF. Negative binomial and mixed poisson regression. Can. J. Stat. 1987;15:209–25.

2. Venables WN, Ripley BD. Modern Applied Statistics with S. New York, NY: Springer New York; 2002.

3. Robinson MD, Smyth GK. Small-sample estimation of negative binomial dispersion, with applications to SAGE data. Biostatistics. 2007;9:321–32.

4. Nelder JA. Quasi-likelihood and pseudo-likelihood are not the same thing. J. Appl. Stat. Taylor & Francis Group; 2000;27:1007–11.

5. Si Y, Liu P. An Optimal Test with Maximum Average Power While Controlling FDR with Application to RNA-Seq Data. Biometrics. 2013;69:594–605.
