# Supplementary material for: Evaluation of logistic regression models and effect of covariates for case–control study in RNA-Seq analysis
Source: BMC Bioinformatics. 2017 Feb 6;18:91. doi: 10.1186/s12859-017-1498-y (PMC5294900; doi:10.1186/s12859-017-1498-y)
Supplement: Additional file 2: Table S1. — Type-I error rates of the NB regression from the balanced design with N D=1 = 10. Mean: The mean expression values in cases and controls, Disp: Dispersion, NB: Negative Binomial regression, MLD: Maximum likelihood estimated dispersion, QLD: Quasi-likelihood estimated dispersion, TD: True dispersion specified in the simulation. (DOCX 55 kb) [file 12859_2017_1498_MOESM2_ESM.docx]

**Table S1**. Type-I error rates of the NB regression from the balanced design with *N_D=1_*=10

|  |  | Alpha = 0.05 | | | Alpha = 0.01 | | |
| --- | --- | --- | --- | --- | --- | --- | --- |
| Mean | Disp | NB_MLD | NB_QLD | NB_TD | NB_MLD | NB_QLD | NB_TD |
| 50 | 0.01 | 0.066 | 0.066 | 0.067 | 0.021 | 0.02 | 0.02 |
| 50 | 0.1 | 0.07 | 0.071 | 0.071 | 0.019 | 0.019 | 0.02 |
| 50 | 0.5 | 0.08 | 0.08 | 0.08 | 0.027 | 0.027 | 0.027 |
| 50 | 1 | 0.085 | 0.085 | 0.085 | 0.03 | 0.03 | 0.03 |
| 1000 | 0.01 | 0.066 | 0.066 | 0.066 | 0.018 | 0.018 | 0.018 |
| 1000 | 0.1 | 0.068 | 0.068 | 0.068 | 0.021 | 0.021 | 0.021 |
| 1000 | 0.5 | 0.077 | 0.077 | 0.077 | 0.024 | 0.024 | 0.024 |
| 1000 | 1 | 0.094 | 0.094 | 0.094 | 0.032 | 0.032 | 0.032 |
