# Supplementary figures and images for: Evaluation of logistic regression models and effect of covariates for case–control study in RNA-Seq analysis
Source: BMC Bioinformatics. 2017 Feb 6;18:91. doi: 10.1186/s12859-017-1498-y (PMC5294900; doi:10.1186/s12859-017-1498-y)

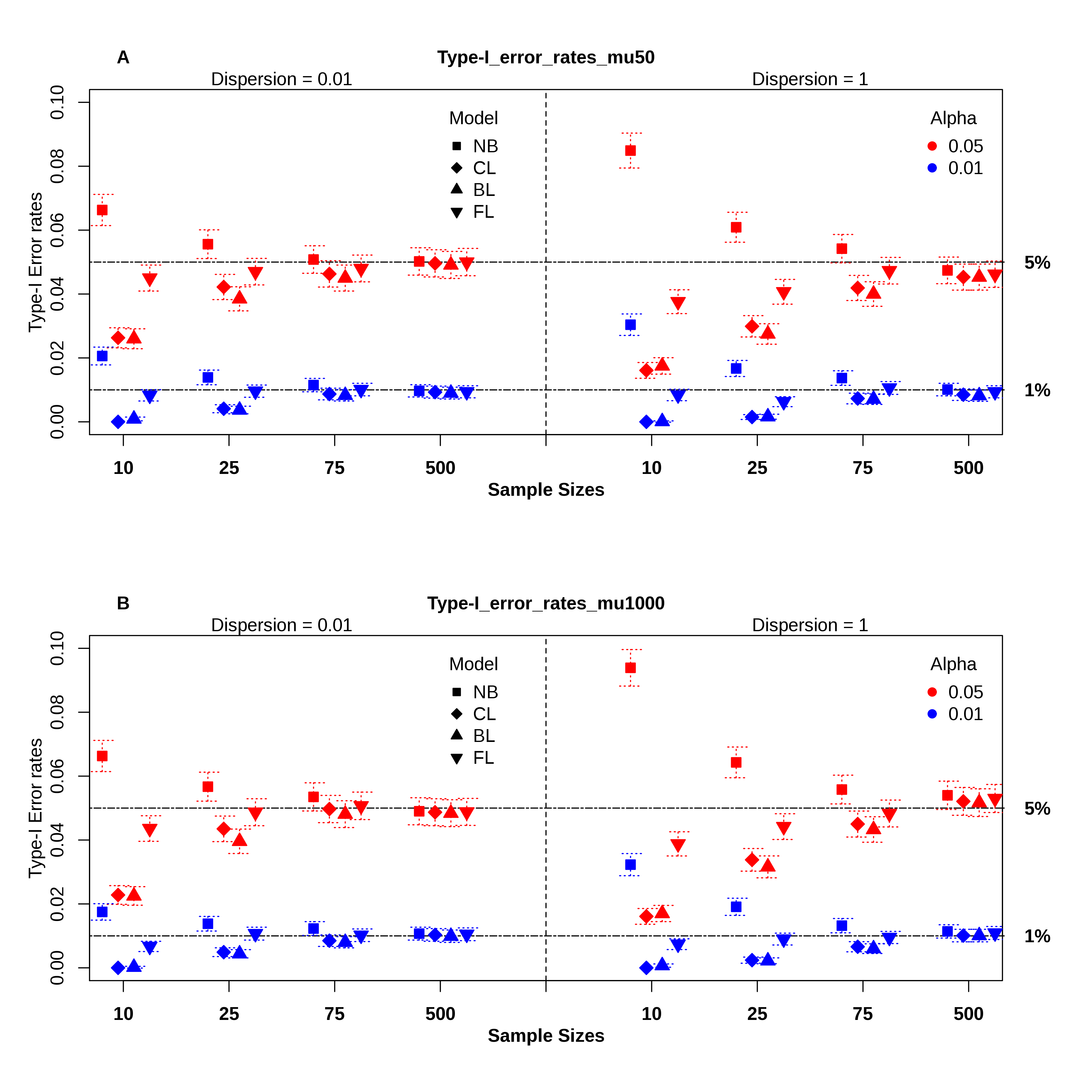

Supplement: Additional file 3: Figure S1. — Type-I error rates of regression methods from the balanced design. Type-I error rates of the Negative Binomial with true dispersion (NB), Classic Logistic (CL), Bayes Logistic (BL), and Firth’s Logistic (FL) regressions at alpha levels of 0.05 and 0.01 are shown. The black dotted horizontal lines represent 5 and 1% of Type-I error rates. Dispersion values (ϕ = 0.01 and 1) are separated by black dotted vertical lines. Four values of the number of cases (10, 25, 75 and 500) are placed within each dispersion value. Dotted lines within each symbol imply 95% confidence interval. Figure S1 (A): The figure presents the Type-I error rates when μ = 50. Figure S1 (B): This figure shows the Type-I error rates when μ = 1000. (PNG 399 kb) [file 12859_2017_1498_MOESM3_ESM.png]

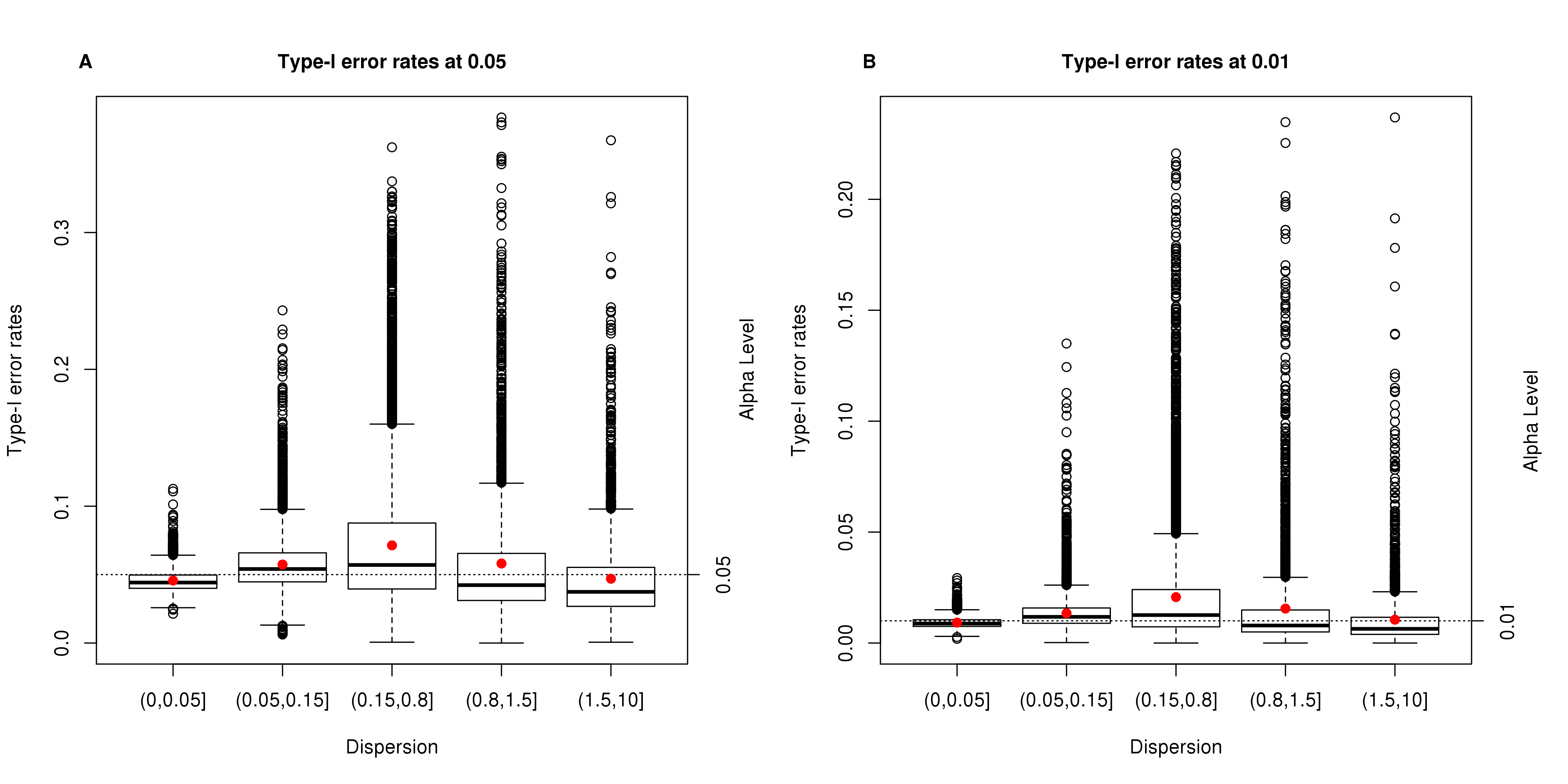

Supplement: Additional file 6: Figure S2. — Type-I error rates from DESeq2 analysis of the permuted HD data. This contains Type-I error rates from DESeq2 (negative binomial model) analysis of the permuted HD data at alpha levels of 0.05 and 0.01. Each black empty dot represents Type-I error rate of a gene. The red dots denote average values of Type-I error rates in each category of dispersion groups. The black dotted horizontal lines are our alpha levels. Figure S2 (A) shows Type-I error rates of all genes at alpha level of 0.05. Figure S2 (B) displays Type-I error rates of all genes at alpha level of 0.01. (PNG 309 kb) [file 12859_2017_1498_MOESM6_ESM.png]

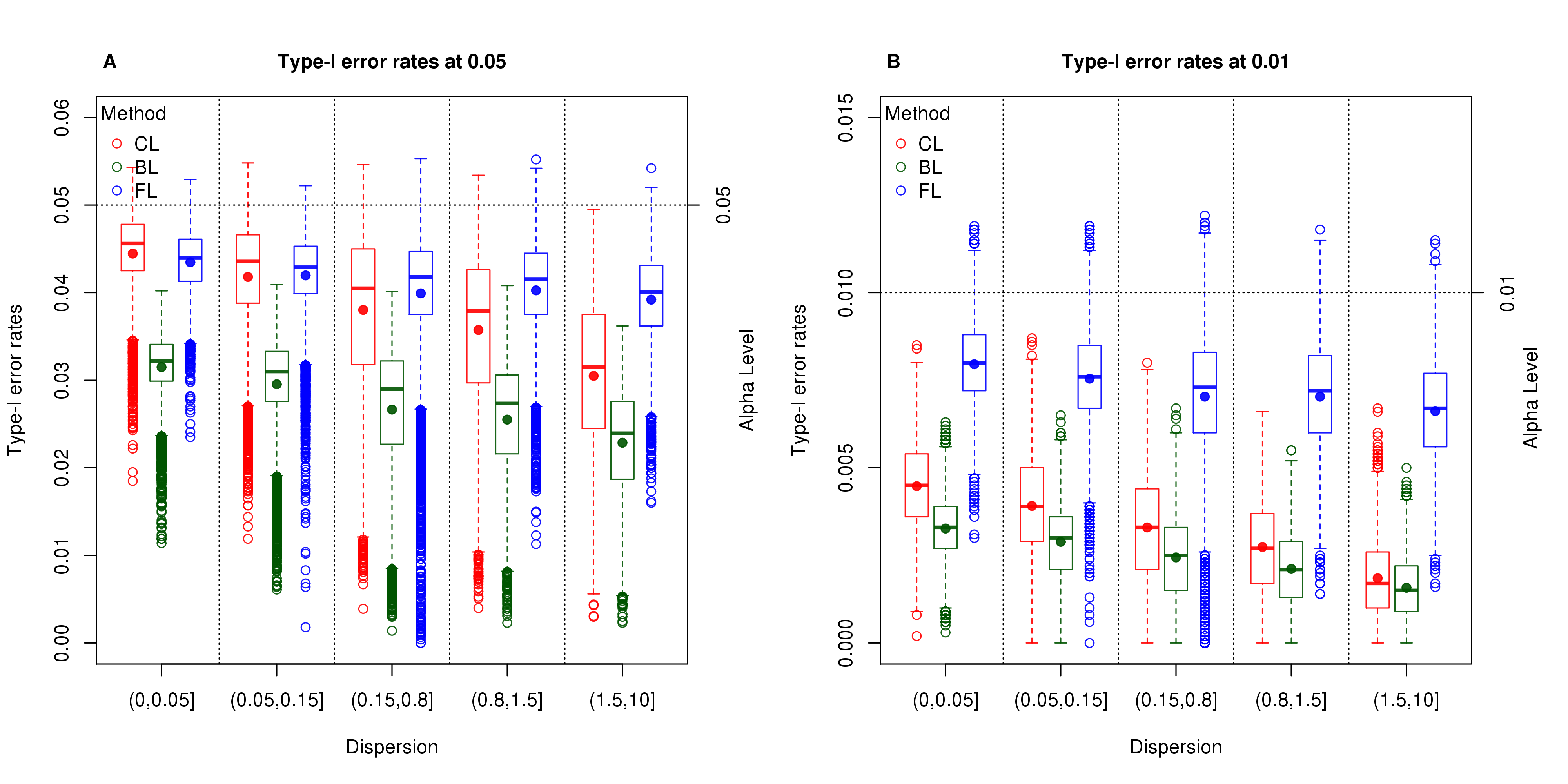

Supplement: Additional file 7: Figure S3. — Type-I error rates from logistic model analyses of the permuted HD data. Figure S3 contains Type-I error rates from Classical Logistic (CL), Bayes Logistic (BL), Firth’s Logistic (FL) regressions of the permuted HD data at alpha levels of 0.05 and 0.01. Each empty dot represents Type-I error rate of a gene. The dots filled with colors inside of boxes denote average values of Type-I error rates in each category of dispersion groups. The black dotted horizontal lines are our alpha levels. Figure S3 (A) shows Type-I error rates of all genes at alpha level of 0.05. Figure S3 (B) displays Type-I error rates of all genes at alpha level of 0.01. (PNG 470 kb) [file 12859_2017_1498_MOESM7_ESM.png]

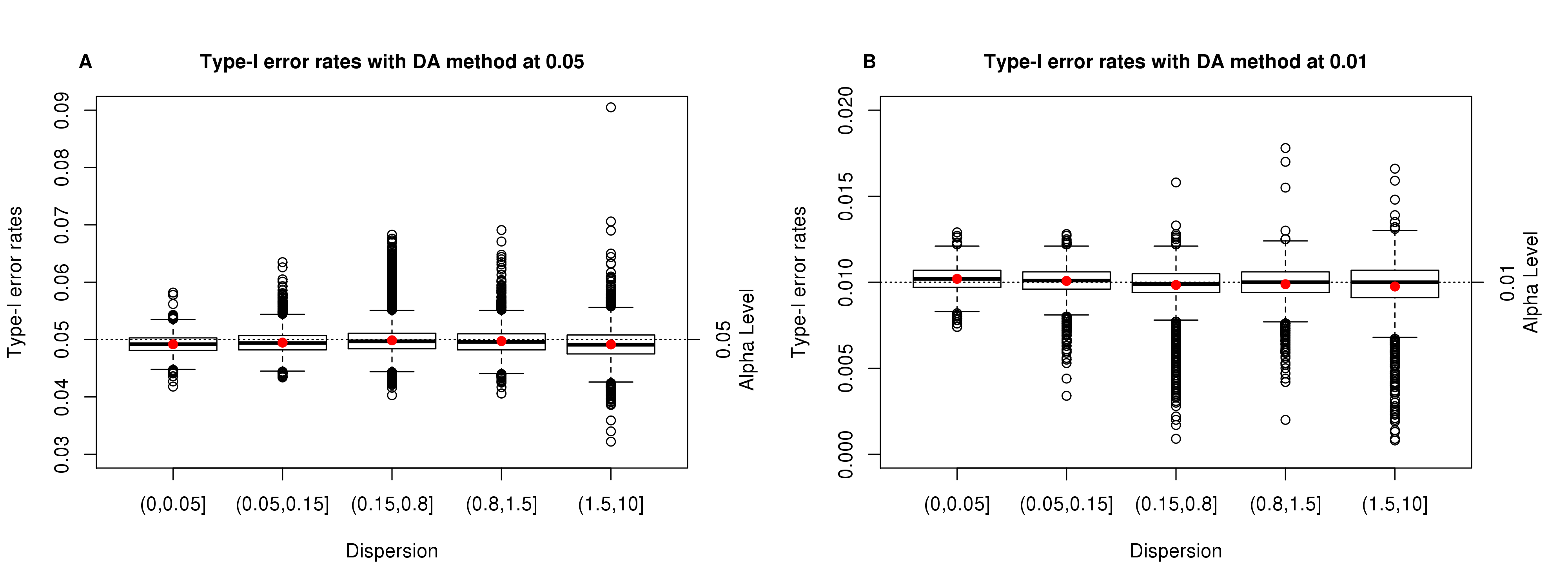

Supplement: Additional file 8: Figure S4. — Type-I error rates from DESeq2 analysis with the DA method using the permuted HD data. Figure S4 contains Type-I error rates from DESeq2 (negative binomial model) analysis with DA method of the permuted HD data at alpha levels of 0.05 and 0.01. Each black empty dot represents Type-I error rate of a gene. The red dots denote average values of Type-I error rates in each category of dispersion groups. The black dotted horizontal lines are our alpha levels. Figure S4 (A) summarizes Type-I error rates of all genes with DA method at alpha level of 0.05. Figure S4 (B) displays Type-I error rates of all genes with DA method at alpha level of 0.01. (PNG 223 kb) [file 12859_2017_1498_MOESM8_ESM.png]

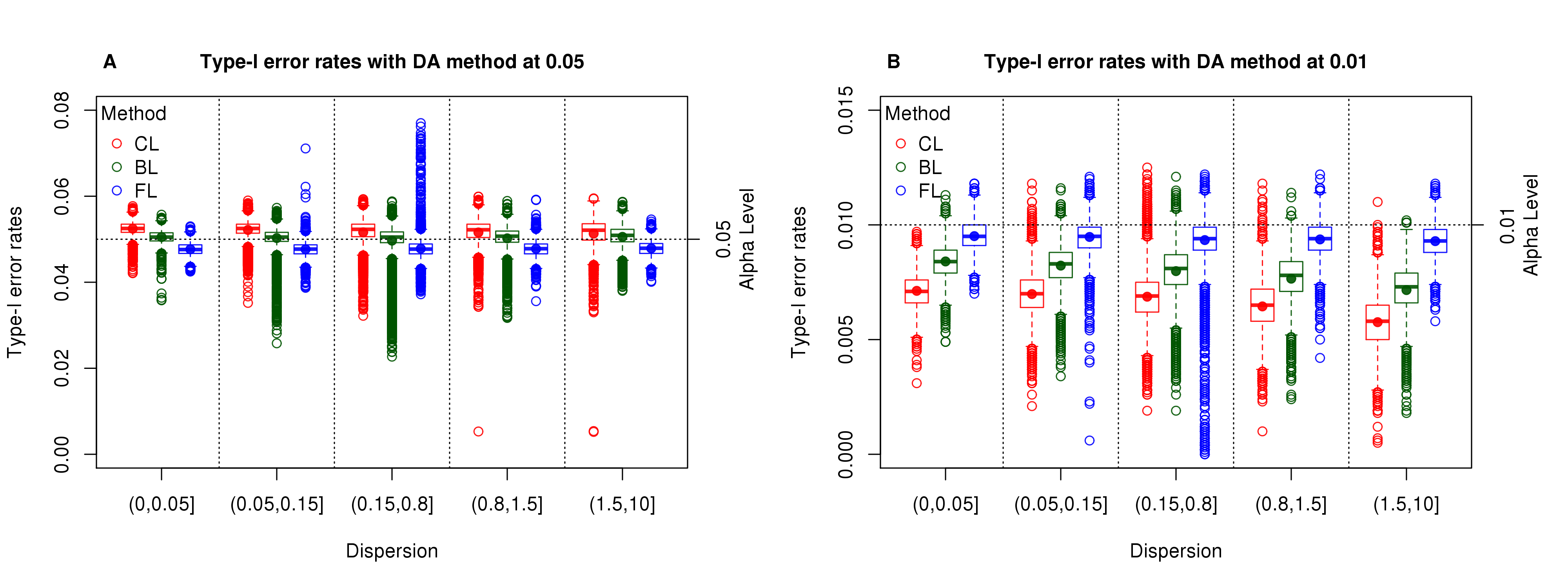

Supplement: Additional file 9: Figure S5. — Type-I error rates from logistic model analyses with the DA method using the permuted HD data. Figure S5 presents Type-I error rates from Classical Logistic (CL), Bayes Logistic (BL), Firth’s Logistic (FL) regressions with the DA method of the permuted HD data at alpha levels of 0.05 and 0.01. Each empty dot represents Type-I error rate of a gene. The dots filled with colors inside of boxes denote average values of Type-I error rates in each category of dispersion groups. The black dotted horizontal lines are our alpha levels. Figure S5 (A) shows Type-I error rates of all genes with DA method at alpha level of 0.05. Figure S5 (B) represents Type-I error rates of all genes with DA method at alpha level of 0.01. (PNG 453 kb) [file 12859_2017_1498_MOESM9_ESM.png]

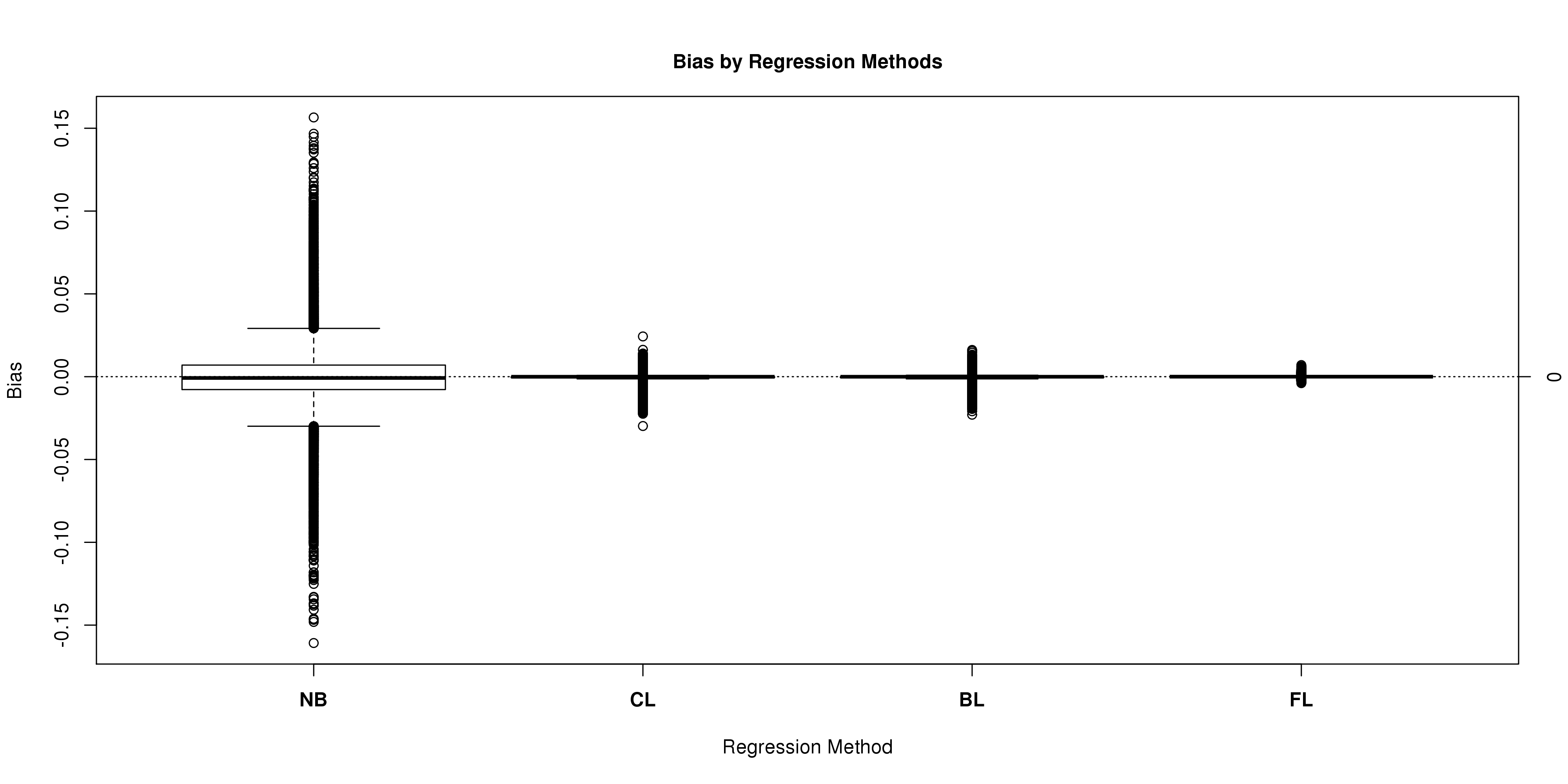

Supplement: Additional file 10: Figure S6. — Bias from regression methods using the permuted HD data with μ g > 3. Figure S6 contains bias from Negative Binomial regression using DESeq2, Classical Logistic regression (CL), Bayes Logistic regression (BL), and Firth’s Logistic regression (FL). Each black empty dot represents the bias of a gene. The black dotted horizontal line is no bias point. The bias of each gene is calculated using effect sizes of 10,000 permutations. (PNG 53 kb) [file 12859_2017_1498_MOESM10_ESM.png]

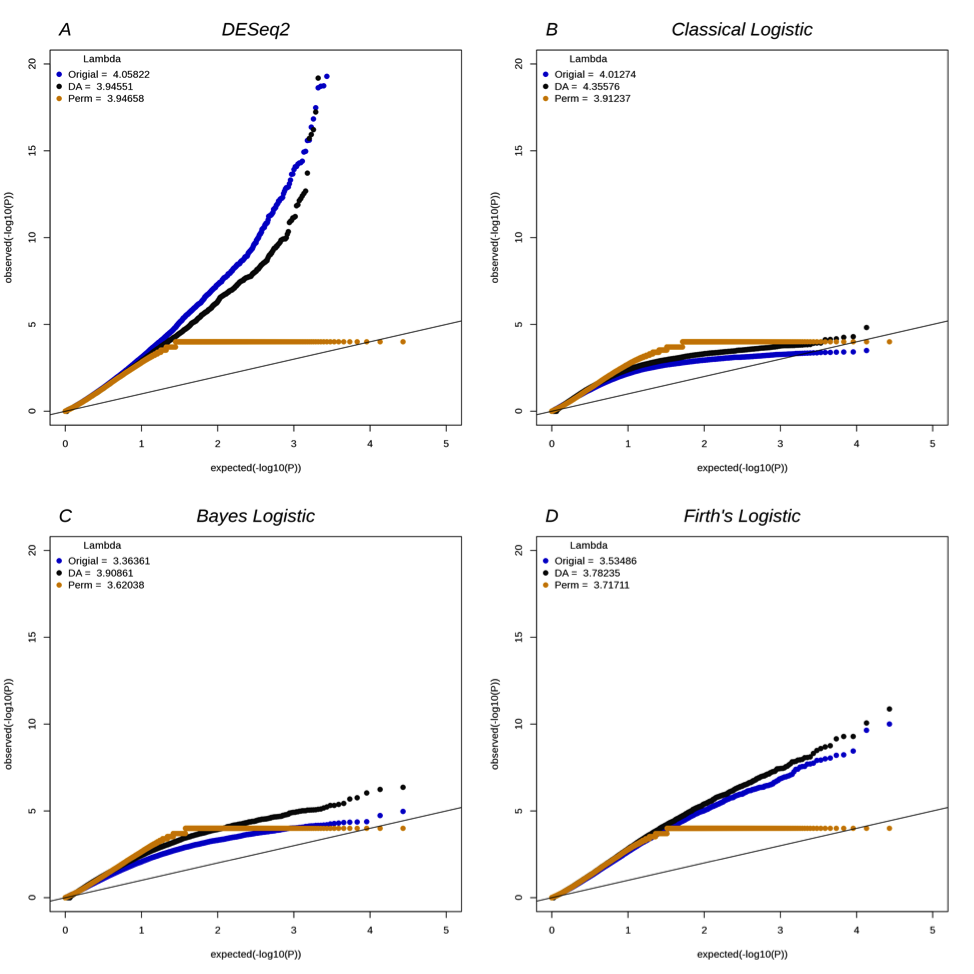

Supplement: Additional file 11: Figure S7. — Q-Q plots of the HD Analyses. Figure S7 exhibits the Q-Q plots from the HD analysis adjusting for age at death and RIN from DESeq2 (A), and Classical (B), Bayes (C), and Firth’s (D) Logistic regressions. Each regression method contains three different ways of calculating p-values (Original, DA, and Perm). “Original” p-values (Blue dots) are estimated from asymptotic distribution. “DA” p-values (Black dots) are evaluated from data adaptive asymptotic distribution using 1,000 permutations. “Perm” p-values (Yellow dots) are calculated using 10,000 permutations. (PNG 157 kb) [file 12859_2017_1498_MOESM11_ESM.png]

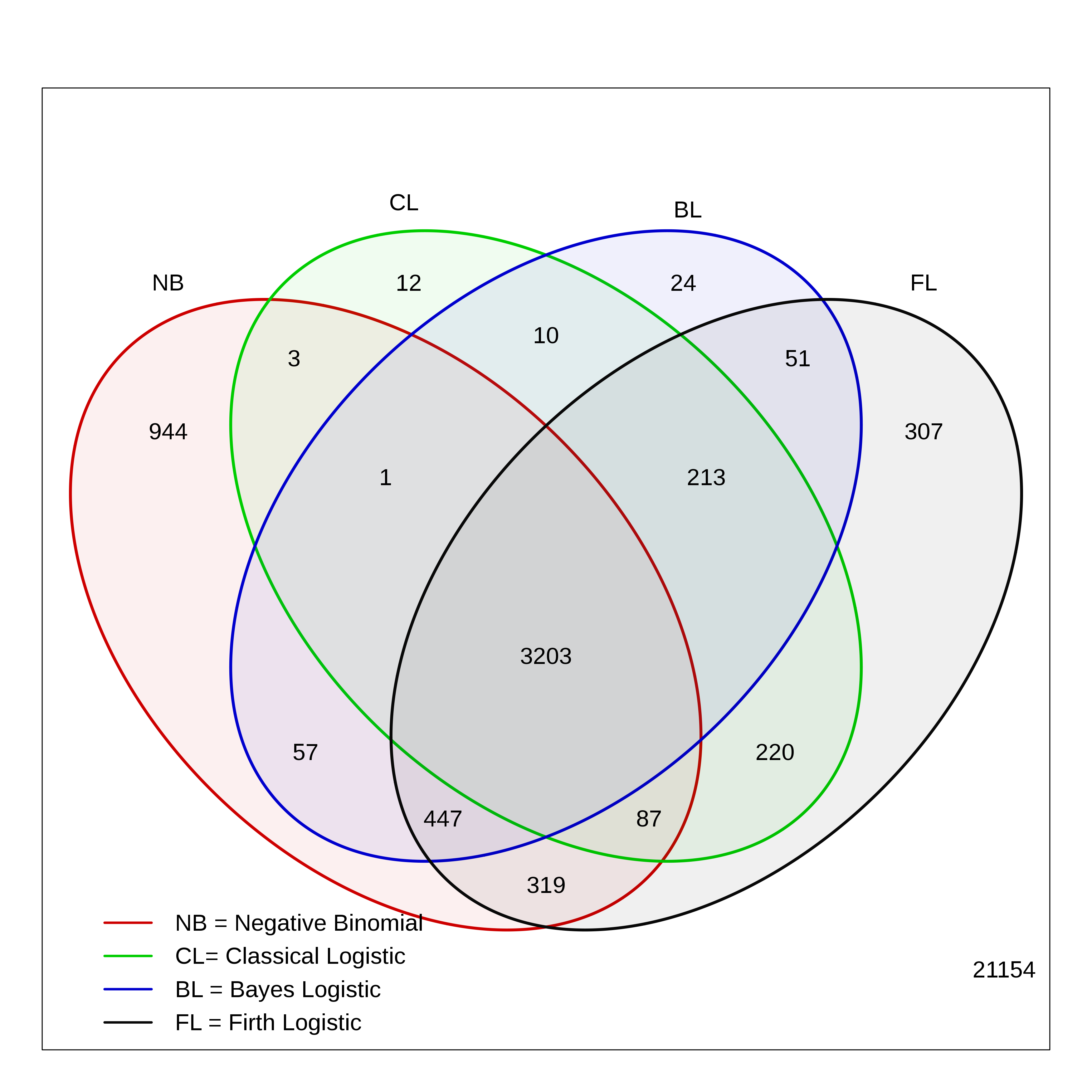

Supplement: Additional file 12: Figure S8. — Venn diagram of HD analysis results using DA method. Each colored circle represents a different regression method. The numbers inside of the circles are the number of genes significant at FDR 0.05 based on p-values adjusted using the Data Adaptive (DA) method. There were 3,203 significant genes in common across all the methods. The FL identified the largest number of significant genes compared to CL and BL. The NB independently identified 944 genes. (PNG 474 kb) [file 12859_2017_1498_MOESM12_ESM.png]
