# Supplementary material for: Evaluation of logistic regression models and effect of covariates for case–control study in RNA-Seq analysis
Source: BMC Bioinformatics. 2017 Feb 6;18:91. doi: 10.1186/s12859-017-1498-y (PMC5294900; doi:10.1186/s12859-017-1498-y)
Supplement: Additional file 4: Table S2. — Type-I error rates of regression methods from the unbalanced design with μ D=0 = 1000. Alpha: Significance levels, N D=1: The number of cases, N D=0: The number of controls, Disp: Dispersion, NB: Negative binomial regression with true dispersion, CL: Classical logistic regression, BL: Bayes logistic regression, FL: Firth’s logistic regression. (DOCX 65 kb) [file 12859_2017_1498_MOESM4_ESM.docx]

**Table S2**. Type-I error rates of regression methods from the unbalanced design with *μ_D=0_* =1000

| Alpha | *N_D=1_* | *N_D=0_* | Disp | NB | CL | BL | FL |
| --- | --- | --- | --- | --- | --- | --- | --- |
| 0.05 | 10 | 20 | 0.01 | 0.059 | 0.032 | 0.027 | 0.045 |
|  | 10 | 20 | 1 | 0.081 | 0.023 | 0.021 | 0.042 |
|  | 10 | 40 | 0.01 | 0.055 | 0.040 | 0.033 | 0.047 |
|  | 10 | 40 | 1 | 0.070 | 0.031 | 0.028 | 0.044 |
|  | 25 | 50 | 0.01 | 0.058 | 0.049 | 0.045 | 0.052 |
|  | 25 | 50 | 1 | 0.062 | 0.037 | 0.033 | 0.048 |
|  | 25 | 100 | 0.01 | 0.055 | 0.048 | 0.044 | 0.050 |
|  | 25 | 100 | 1 | 0.059 | 0.038 | 0.036 | 0.045 |
| 0.01 | 10 | 20 | 0.01 | 0.015 | 0.000 | 0.001 | 0.008 |
|  | 10 | 20 | 1 | 0.024 | 0.001 | 0.001 | 0.007 |
|  | 10 | 40 | 0.01 | 0.012 | 0.002 | 0.002 | 0.007 |
|  | 10 | 40 | 1 | 0.022 | 0.003 | 0.003 | 0.007 |
|  | 25 | 50 | 0.01 | 0.014 | 0.007 | 0.006 | 0.010 |
|  | 25 | 50 | 1 | 0.016 | 0.004 | 0.003 | 0.008 |
|  | 25 | 100 | 0.01 | 0.012 | 0.008 | 0.007 | 0.011 |
|  | 25 | 100 | 1 | 0.013 | 0.005 | 0.004 | 0.008 |
