# Supplementary material for: Evaluation of logistic regression models and effect of covariates for case–control study in RNA-Seq analysis
Source: BMC Bioinformatics. 2017 Feb 6;18:91. doi: 10.1186/s12859-017-1498-y (PMC5294900; doi:10.1186/s12859-017-1498-y)
Supplement: Additional file 5: Table S3. — Empirical power of NB regression from the balanced design with N D=1 = 10 and log2fc = 0.3. Mean: The mean expression values in cases and controls, Disp: Dispersion, NB: Negative Binomial regression, MLD: Maximum likelihood estimated dispersion, QLD: Quasi-likelihood estimated dispersion, TD: True dispersion specified in the simulation. (DOCX 56 kb) [file 12859_2017_1498_MOESM5_ESM.docx]

**Table S3**. Empirical power of NB regression from the balanced design with *N_D=1_*=10 and log2fc=0.3

|  |  | Alpha = 0.05 | | | Alpha = 0.01 | | |
| --- | --- | --- | --- | --- | --- | --- | --- |
| Mean | Disp | NB_MLD | NB_QLD | NB_TD | NB_MLD | NB_QLD | NB_TD |
| 50 | 0.01 | 0.781 | 0.78 | 0.781 | 0.503 | 0.503 | 0.504 |
| 50 | 0.1 | 0.261 | 0.262 | 0.262 | 0.096 | 0.096 | 0.096 |
| 50 | 0.5 | 0.089 | 0.089 | 0.089 | 0.021 | 0.021 | 0.021 |
| 50 | 1 | 0.075 | 0.074 | 0.074 | 0.014 | 0.014 | 0.014 |
| 1000 | 0.01 | 0.989 | 0.989 | 0.989 | 0.939 | 0.94 | 0.94 |
| 1000 | 0.1 | 0.267 | 0.267 | 0.267 | 0.089 | 0.089 | 0.089 |
| 1000 | 0.5 | 0.093 | 0.093 | 0.093 | 0.024 | 0.024 | 0.024 |
| 1000 | 1 | 0.063 | 0.063 | 0.063 | 0.014 | 0.014 | 0.014 |
