# Supplementary material for: Evaluation of logistic regression models and effect of covariates for case–control study in RNA-Seq analysis
Source: BMC Bioinformatics. 2017 Feb 6;18:91. doi: 10.1186/s12859-017-1498-y (PMC5294900; doi:10.1186/s12859-017-1498-y)
Supplement: Additional file 13: Table S4. — Top 10 significant genes from DESeq2 among genes not significant in logistic regressions. Mean.Exp.Case: Normalized mean expression value in cases, Mean.Exp.Cont: Normalized mean expression value in controls, Disp: Dispersion, NB.Pval: P-values from negative binomial regression with true dispersion, CL.Pval: P-values from classical logistic regression, BL.Pval: P-values from Bayes logistic regression, FL.Pval: P-values from Firth’s logistic regression. (DOCX 53 kb) [file 12859_2017_1498_MOESM13_ESM.docx]

**Table S4**. Top 10 significant genes from DESeq2 among genes not significant in logistic regressions.

| Gene | Mean.Exp.  Case | Mean.Exp.  Cont | Disp | NB.Pval | CL.Pval | BL.Pval | FL.Pval |
| --- | --- | --- | --- | --- | --- | --- | --- |
| *RP11-115J23.1* | 2.8 | 0.4 | 2.19 | 9.67E-06 | 0.019 | 0.011 | 0.012 |
| *CTD-2281E23.3* | 0.6 | 2.5 | 1.20 | 3.26E-05 | 0.028 | 0.016 | 0.020 |
| *LL22NC03-104C7.1* | 1.1 | 7.3 | 1.54 | 3.42E-05 | 0.036 | 0.014 | 0.009 |
| *CEACAM3* | 2.9 | 0.4 | 2.53 | 5.62E-05 | 0.044 | 0.020 | 0.016 |
| *RP11-351I21.6* | 1.7 | 11.8 | 1.58 | 6.72E-05 | 0.043 | 0.022 | 0.023 |
| *LINC00310* | 29.7 | 9.9 | 0.73 | 9.91E-05 | 0.025 | 0.013 | 0.010 |
| *RP5-850O15.3* | 0.4 | 2.8 | 1.55 | 1.06E-04 | 0.020 | 0.018 | 0.010 |
| *RP11-554A11.9* | 15.4 | 37.8 | 0.55 | 1.70E-04 | 0.014 | 0.009 | 0.009 |
| *GK3P* | 8.1 | 15.9 | 0.65 | 1.75E-04 | 0.020 | 0.013 | 0.014 |
| *S100A11* | 568.0 | 266.0 | 0.44 | 2.43E-04 | 0.019 | 0.015 | 0.013 |
