# Supplementary material for: Evaluation of logistic regression models and effect of covariates for case–control study in RNA-Seq analysis
Source: BMC Bioinformatics. 2017 Feb 6;18:91. doi: 10.1186/s12859-017-1498-y (PMC5294900; doi:10.1186/s12859-017-1498-y)
Supplement: Additional file 15: Table S6. — Type-I error rates of the NB regression from the balanced design with N D=1 = 10 and μ = 1000. Disp: Dispersion, CovOR: Odds ratios between covariates and case–control status, Ncov: The number of covariates in a model, NB: Negative binomial regression, MLD: Maximum likelihood estimated Dispersion, QLD: Quasi-likelihood estimated Dispersion, TD: The dispersion is used for the sampling. (DOCX 59 kb) [file 12859_2017_1498_MOESM15_ESM.docx]

**Table S6**. Type-I error rates of the NB regression from balanced design with *N_D=1_*=10 and *μ*=1000

|  |  |  | Alpha =0.05 | | | Alpha = 0.01 | | |
| --- | --- | --- | --- | --- | --- | --- | --- | --- |
| Disp | CovOR | Ncov | NB_MLD | NB_QLD | NB_TD | NB_MLD | NB_QLD | NB_TD |
| 0.01 | 1.2 | 1 | 0.0712 | 0.0711 | 0.0710 | 0.0230 | 0.0230 | 0.0228 |
| 0.01 | 1.2 | 5 | 0.0754 | 0.0755 | 0.0756 | 0.0259 | 0.0259 | 0.0259 |
| 0.01 | 5 | 1 | 0.0611 | 0.0611 | 0.0611 | 0.0184 | 0.0184 | 0.0184 |
| 0.01 | 5 | 5 | 0.0797 | 0.0798 | 0.0797 | 0.0237 | 0.0237 | 0.0238 |
| 1 | 1.2 | 1 | 0.1026 | 0.1026 | 0.1026 | 0.0358 | 0.0358 | 0.0358 |
| 1 | 1.2 | 5 | 0.1506 | 0.1508 | 0.1509 | 0.0670 | 0.0671 | 0.0671 |
| 1 | 5 | 1 | 0.1021 | 0.1021 | 0.1021 | 0.0366 | 0.0367 | 0.0367 |
| 1 | 5 | 5 | 0.1419 | 0.1420 | 0.1418 | 0.0611 | 0.0610 | 0.0610 |
