# Supplementary material for: Evaluation of logistic regression models and effect of covariates for case–control study in RNA-Seq analysis
Source: BMC Bioinformatics. 2017 Feb 6;18:91. doi: 10.1186/s12859-017-1498-y (PMC5294900; doi:10.1186/s12859-017-1498-y)
Supplement: Additional file 16: Table S7. — Bias with covariate models from the balanced design of N D=1 = 10 and μ D=0 = 1000. Disp: Dispersion, CovOR: Odds ratios between covariates and case–control status, Ncov: The number of covariates in a model, NB_TD: Negative binomial regression with the dispersion is used for the sampling, FL: Firth’s logistic regression. (DOCX 47 kb) [file 12859_2017_1498_MOESM16_ESM.docx]

**Table S7**. Bias with covariate models from balanced design of *N_D=1_*=10 and *μ_D=0_*=1000

| Disp | CovOR | Ncov | Bias of beta coefficient | |
| --- | --- | --- | --- | --- |
|  |  |  | NB_TD | FL |
| 0.01 | 1.2 | 1 | 4.03E-04 | 3.10E-05 |
| 0.01 | 1.2 | 5 | 2.95E-04 | 5.60E-05 |
| 0.01 | 5 | 1 | -7.04E-05 | -1.94E-05 |
| 0.01 | 5 | 5 | -6.36E-04 | -9.04E-05 |
| 1 | 1.2 | 1 | 4.44E-03 | 3.99E-06 |
| 1 | 1.2 | 5 | 6.09E-03 | 7.92E-06 |
| 1 | 5 | 1 | 1.31E-02 | -1.58E-06 |
| 1 | 5 | 5 | 4.03E-02 | -6.07E-06 |
