# Supplementary material for: Evaluation of logistic regression models and effect of covariates for case–control study in RNA-Seq analysis
Source: BMC Bioinformatics. 2017 Feb 6;18:91. doi: 10.1186/s12859-017-1498-y (PMC5294900; doi:10.1186/s12859-017-1498-y)
Supplement: Additional file 17: Table S8. — Type-I error rates of the NB regression from the balanced design with N D=1 = 10, μ D=0 = 1000, and log2fc = 0.3. Disp: Dispersion, CovOR: Odds ratios between covariates and case–control status, Ncov: The number of covariates in a model, NB: Negative binomial regression, MLD: Maximum likelihood estimated Dispersion, QLD: Quasi-likelihood estimated Dispersion, TD: The dispersion is used for the sampling. (DOCX 59 kb) [file 12859_2017_1498_MOESM17_ESM.docx]

**Table S8**. Type-I error rates of the NB regression from balanced design with *N_D=1_*=10, *μ_D=0_*=1000, and log2fc=0.3

|  |  |  | Alpha = 0.05 | | | Alpha = 0.01 | | |
| --- | --- | --- | --- | --- | --- | --- | --- | --- |
| Disp | CovOR | Ncov | NB_MLD | NB_QLD | NB_TD | NB_MLD | NB_QLD | NB_TD |
| 0.01 | 1.2 | 1 | 0.979 | 0.979 | 0.979 | 0.887 | 0.887 | 0.886 |
| 0.01 | 1.2 | 5 | 0.920 | 0.920 | 0.919 | 0.741 | 0.742 | 0.743 |
| 0.01 | 5 | 1 | 0.962 | 0.962 | 0.962 | 0.845 | 0.846 | 0.846 |
| 0.01 | 5 | 5 | 0.712 | 0.712 | 0.712 | 0.457 | 0.457 | 0.454 |
| 1 | 1.2 | 1 | 0.069 | 0.069 | 0.069 | 0.017 | 0.017 | 0.017 |
| 1 | 1.2 | 5 | 0.046 | 0.046 | 0.046 | 0.009 | 0.009 | 0.009 |
| 1 | 5 | 1 | 0.070 | 0.070 | 0.070 | 0.015 | 0.015 | 0.015 |
| 1 | 5 | 5 | 0.048 | 0.048 | 0.048 | 0.012 | 0.012 | 0.012 |
